# Humanin Mitigates Aβ‐Induced Retinal Pigment Epithelium Injury via AMPK‐Beclin1‐Dependent Mitophagy

**DOI:** 10.1111/acel.70601

**Published:** 2026-06-23

**Authors:** Ha Young Jang, Suyeon Choi, Soo‐Jin Kim, Sooyun Kim, Dong Hyun Jo, Tae Geol Lee, Kyu‐Sang Park, Jeong Hun Kim

**Affiliations:** ^1^ Fight Against Angiogenesis‐Related Blindness (FARB) Laboratory, Biomedical Research Institute Seoul National University Hospital Seoul Republic of Korea; ^2^ Global Excellence Center for Gene & Cell Therapy (GEC‐GCT) Seoul National University Hospital Seoul Republic of Korea; ^3^ Department of Veterinary Nursing, College of Health Sciences Wonkwang University Iksan Republic of Korea; ^4^ Department of Physiology and Global Medical Science Yonsei University Wonju College of Medicine Wonju Republic of Korea; ^5^ Organelle Medicine Research Center Yonsei University Wonju College of Medicine Wonju Republic of Korea; ^6^ Department of Anatomy & Cell Biology Seoul National University College of Medicine Seoul Republic of Korea; ^7^ Center of Nano‐Bio Measurement Korea Research Institute of Standards and Science (KRISS) Daejeon Korea; ^8^ Department of Biomedical Sciences & Ophthalmology Seoul National University College of Medicine Seoul Republic of Korea; ^9^ Institute of Reproductive Medicine and Population Seoul National University College of Medicine Seoul Republic of Korea

**Keywords:** age‐related macular degeneration, amyloid beta, humanin, mitochondrial dysfunction, retinal pigment epithelium

## Abstract

Amyloid beta (Aβ), a key component of drusen in age‐related macular degeneration (AMD), induces oxidative stress, mitochondrial dysfunction, and degeneration in the retinal pigment epithelium (RPE), contributing to progressive vision loss in the elderly. We investigated the protective role of Humanin (HN), a mitochondria‐derived peptide with known neuroprotective effects in Aβ‐related neurodegenerative diseases, in retinal pathology induced by subretinal injection of FITC‐labeled Aβ. HN enhanced the clearance of Aβ‐accumulated mitochondria in the RPE while preserving retinal function and RPE barrier integrity. In ARPE‐19 cells, HN activated AMP‐activated protein kinase (AMPK), leading to phosphorylation of ULK1 and Beclin1, which promoted the interaction between Beclin1 and Parkin and their translocation to mitochondria. This process facilitated the removal of Aβ‐accumulated mitochondria in the RPE. Our results demonstrate that targeting mitophagy in the RPE with HN may offer a promising therapeutic strategy for AMD.

## Graphical abstract

Humanin (HN) enhanced the clearance of Aβ‐accumulated mitochondria in the RPE while preserving retinal function and RPE barrier integrity. HN activated AMP‐activated protein kinase (AMPK), leading to phosphorylation of ULK1 and Beclin1, which promoted the interaction between Beclin1 and Parkin and their translocation to mitochondria.

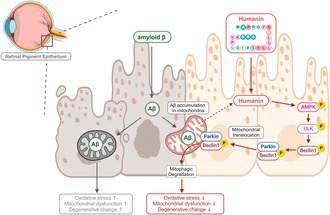

**Abbreviations**
ACC: acetyl‐CoA carboxylase
AD: Alzheimer's disease
AMD: age‐related macular degeneration
AMPK: AMP‐activated protein kinase
Aβ: amyloid beta
Baf.A1: Bafilomycin A1
BSO: (buthioninebuthionine sulfoximine)
CMA: chaperone‐mediated autophagy
ERG: Electroretinography
FCCP: carbonyl cyanide‐4‐(trifluoromethoxy)Phenylhydrazone
FITC: fluorescein isothiocyanate
HN: Humanin
IVT: intravitreal
LAMP1: Lysosomal Associated Membrane Protein 1
OCR: oxygen consumption rate
ORF: open reading frame
RCSs: RPE‐choroid‐scleral complexes
RGCs: retinal ganglion cells
ROS: reactive oxygen species
RPE: retinal pigment epithelium
SR: subretinal
TEER: transepithelial electrical resistance
TFEB: Transcription factor EB
ULK1: unc‐51 like autophagy activating kinase 1

## 1 Introduction

Age‐related macular degeneration (AMD) is a prevalent retinal degenerative disease and the leading cause of vision loss among the elderly population that affects approximately 200 million people globally (Wong et al. 2014). The predominant dry form is characterized by drusen deposits, retinal pigment epithelium (RPE) dysfunction and degeneration, and photoreceptor loss (Chuang et al. 2022). Drusen, which accumulate as extracellular debris beneath the RPE and above Bruch's membrane, play a pivotal role in the clinical manifestation of dry AMD (Luibl et al. 2006). These deposits are composed of diverse proteins associated with inflammation and the innate immune response, including amyloid beta (Aβ) and complement components (Masuzzo et al. 2016; Ratnayaka et al. 2015). Aβ, a potent activator of the complement cascade, is elevated in the degenerative retina and participates in various phases of AMD progression by enhancing inflammatory activity, triggering mitochondrial dysfunction, modifying ribosome function, regulating angiogenesis, influencing RNA splicing, and altering cell structure (Wang et al. 2021). Notably, Aβ can accumulate in cellular compartments such as mitochondria and lysosomes, impairing cellular function. In particular, mitochondrial DNA exhibits a deficiency in DNA repair ability and is vulnerable to oxidative damage caused by Aβ, which induces mitochondrial dysfunction and significantly disrupts cellular respiration, thereby contributing to the deterioration of degenerative diseases such as AMD and Alzheimer's disease (AD) (Biscetti et al. 2017; Nashine et al. 2018; Ohno‐Matsui 2011; Reddy and Oliver 2019). Therefore, the specific and active elimination of Aβ is considered a promising therapeutic strategy for AMD (Ding et al. 2008; Muraleva et al. 2019), given its implicated role in AMD pathogenesis (Mody and Joshi 2023).

Humanin (HN), a mitochondrial‐derived peptide encoded by a 75 bp region in the open reading frame (ORF) of mitochondrial 16S ribosomal RNA, was initially identified as a crucial factor in neuronal survival (Hazafa et al. 2021; Reynolds et al. 2020). HN is endogenously produced in various tissues, including the kidneys, skeletal muscles, brain, heart, and liver (Charununtakorn et al. 2016; Kwon et al. 2020; Liu et al. 2019; Peng et al. 2018). Its effects at the cellular level extend to systemic circulation, with circulating levels reportedly decreasing with age in both mice and humans (Conte et al. 2019; Muzumdar et al. 2009). Although the precise role of HN is not yet fully understood, it exhibits cytoprotective, antioxidant, anti‐inflammatory, and metabolic properties in numerous disease models, including neurodegenerative and retinal diseases, suggesting its potential to alleviate mitochondrial dysfunction (Chai et al. 2014; Yang et al. 2018). The role of HN may be related to age‐dependent accumulation of mitochondrial DNA (mtDNA) damage, including deletions and point mutations, as well as a decrease in mitochondrial quantity and mtDNA copy number in specific organs (Kennedy et al. 2013; Mendelsohn and Larrick 2018). HN induces macroautophagy and chaperone‐mediated autophagy (CMA), contributing to cellular quality control and cytoprotection by directing oxidized proteins to lysosomes (Gong et al. 2018; Kim et al. 2022).

Here, we demonstrate that HN suppresses Aβ‐induced degenerative changes, including retinal dysfunction and disruption of the RPE, which are major pathological features of dry AMD. HN treatment alleviates mitochondrial damage and oxidative stress, concurrently reducing Aβ‐accumulated mitochondria. These protective effects are mediated through AMP‐activated protein kinase (AMPK)‐dependent phosphorylation of unc‐51‐like autophagy activating kinase 1 (ULK1) and Beclin1, which promotes the interaction between Beclin1 and Parkin, leading to their translocation to mitochondria and facilitating clearance of Aβ‐damaged mitochondria. Our findings suggest that HN promotes mitochondrial quality control and may serve as a potential therapeutic strategy for age‐related retinal degeneration, such as AMD.

## 2 Results

### 2.1 HN Alleviates Aβ‐Induced Oxidative Stress, Mitochondrial Dysfunction, and Degenerative Changes in the RPE

**FIGURE 1:**
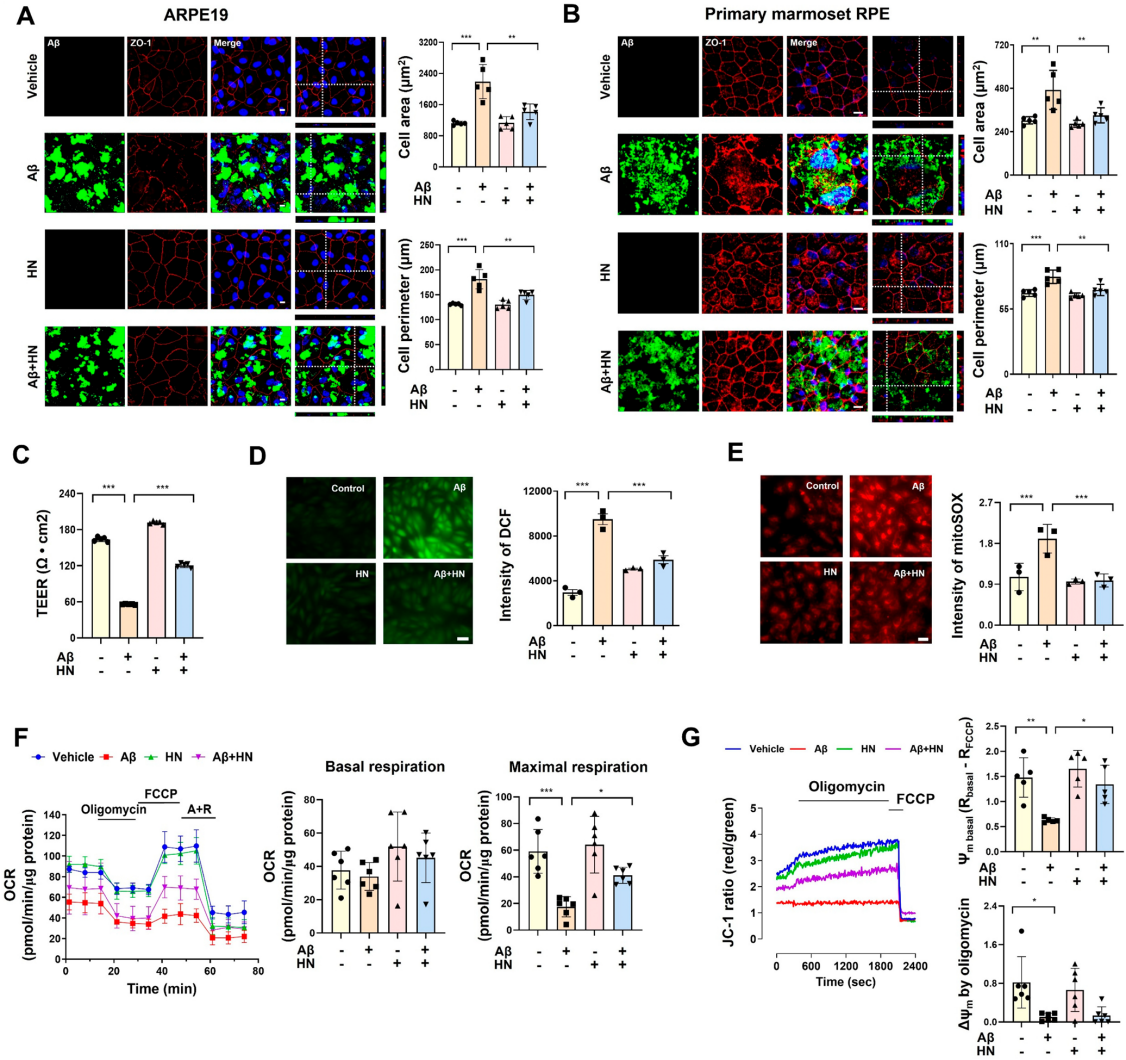
HN mitigated Aβ‐induced oxidative stress, mitochondrial dysfunction, and degenerative changes in RPE cells. (A) Representative confocal images depict disrupted ARPE19 cell morphology, characterized by increased cell size and perimeter due to the entry of FITC‐labeled Aβ. HN treatment restores the morphology to a state similar to that of vehicle‐treated cells. Images and quantification were obtained 24 h after exposure to Aβ and HN. Scale bar, 10 μm. (B) Representative images depict primary marmoset RPE cell morphology similar to ARPE19 cells. Images and quantification were obtained 24 h after exposure to Aβ and HN. Scale bar, 10 μm. (C) Primary marmoset RPE cells differentiated on transwell membranes were exposed to Aβ and HN, and TEER was measured 24 h later. TEER demonstrates that HN preserves tight junction integrity and barrier function, which had been disrupted by Aβ. (D and E) ARPE19 cells were loaded with DCF‐DA (5 μM) or mitoSOX (5 μM) to measure cytosolic (D) or mitochondrial (E) ROS generation, respectively, through fluorescence imaging analysis. (F), The oxygen consumption rate (OCR) was measured in ARPE19 cells using an XF‐96 analyzer, recording baseline level and the effects of oligomycin, FCCP, and antimycin A/rotenone (A + *R*). (G) ARPE19 cells were loaded with JC‐1 (350 nM) and measure the red (Ex/Em: 490/535 nm) and green (540/590 nm) using FlexStation. Mitochondrial membrane potential (ΔΨm) was estimated using the JC‐1‐induced fluorescence ratio of red to green. The number of biologically independent experiments is 5 in (A, B, F, G), 4 in (C), and 3 in (D, E). Data are presented as mean ± SD. *p* values were determined using one‐way ANOVA with Tukey's multiple‐comparison analysis. *p < 0.05, **p < 0.01, and ***p < 0.001. Scale bar, 20 μm.

To investigate the effects of Aβ on mitochondrial dysfunction and oxidative stress, ARPE19 cells and primary marmoset RPE cells were exposed to oligomeric Aβ1‐42. Exogenously treated FITC‐labeled Aβ entered adapted ARPE19 cells, disrupting the intercellular junctions of ZO‐1, a critical tight junction protein necessary for maintaining the structure and integrity of the RPE. This disruption of tight junction led to an increase in both cell area and perimeter. However, treatment with HN mitigated these Aβ‐induced morphological alterations, including the increases in cell area and perimeter (Figure 1A). In primary marmoset RPE cells, Aβ treatment induced multinucleation with nuclear enlargement, in addition to the morphological changes observed in ARPE19 cells, and these alterations were restored by HN treatment (Figure 1B).

In addition to morphological assessments, we measured the substantial transepithelial electrical resistance (TEER) in mature primary marmoset RPE cells to confirm the tight junction integrity and barrier function (Srinivasan et al. 2015). Aβ exposure compromised the TEER value, indicating disrupted barrier function, but this effect was significantly restored by HN co‐treatment (Figure 1C). Aβ increased cytosolic reactive oxygen species (Figure 1D) and mitochondrial superoxide (Figure 1E), both of which were prevented by the cotreatment with HN. As an indicator of mitochondrial activity, oxygen consumption, particularly protonophore (FCCP)‐induced maximal respiration, was markedly deteriorated by Aβ (Figure 1F). HN partially recovered Aβ‐induced suppression in mitochondrial respiratory capacity. The fluorescence ratio of potentiometric dye, JC‐1, which reflects mitochondrial membrane potential, was reduced by Aβ, indicating mitochondrial depolarization. Aβ exposure abolished the hyperpolarizing response to oligomycin due to the accumulation of ATP‐consuming dysfunctional mitochondria (Figure 1G). Consistently, HN restored mitochondrial electrical gradient as well as the hyperpolarizing response to oligomycin. Taken together, HN protects against oxidative stress, mitochondrial dysfunction, and degenerative changes with damaged barrier integrity elicited by pathogenic Aβ exposure.

### 2.2 HN Mitigates Mitochondrial Dysfunction by Enhancing Aβ‐Accumulated Mitochondria Clearance

**FIGURE 2:**
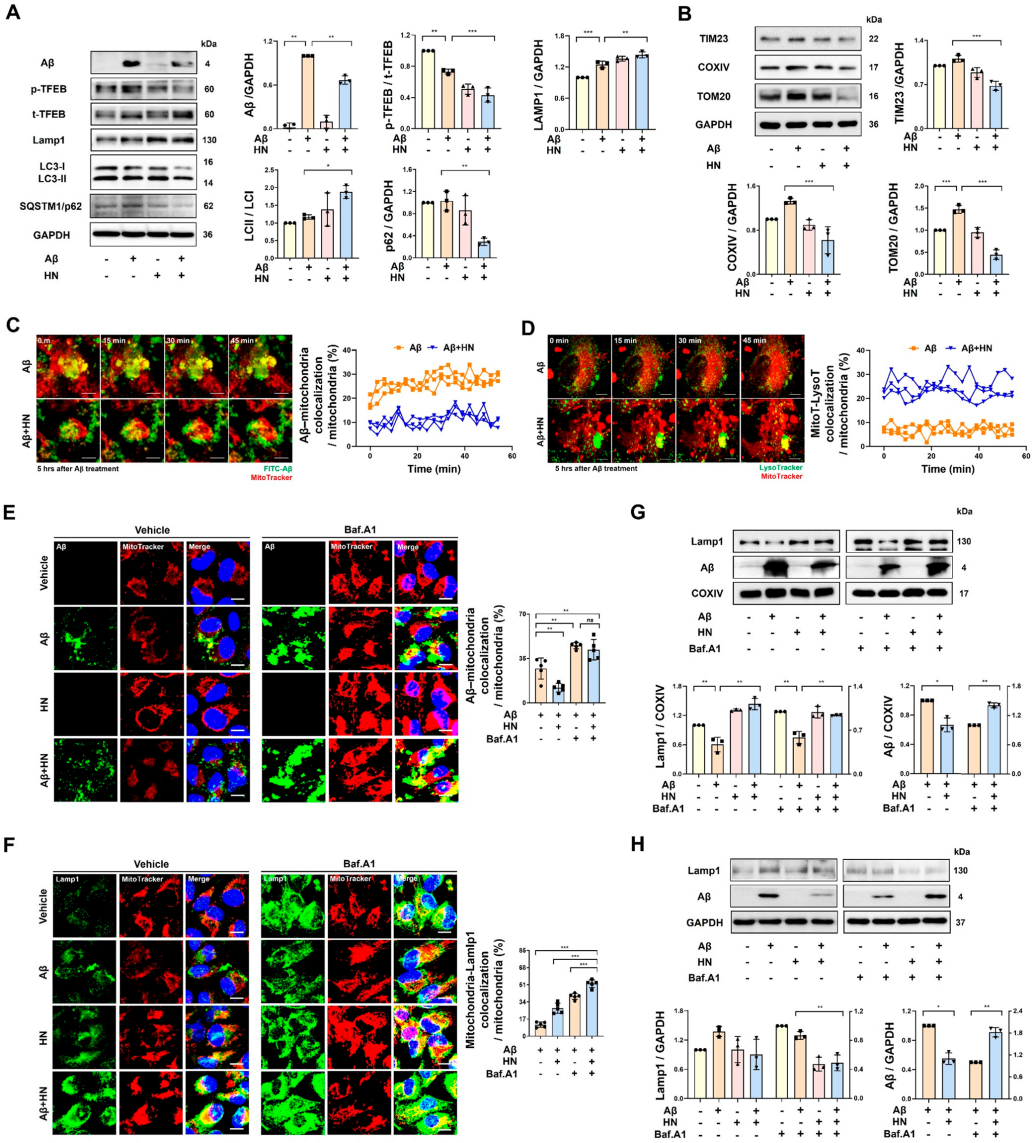
HN reduced the mitochondrial mass by enhancing the removal of Aβ‐accumulated mitochondria. (A, B) Representative immunoblots and quantification show that HN reduces Aβ by regulating lysosomal biogenesis‐related TFEB and autophagic protein LC3, while simultaneously eliminating mitochondrial mass (TIM23, COX IV, TOM20). Proteins were obtained from whole cell lysates of ARPE19 cells 6 h after exposure to Aβ and HN. (C) Dual‐color time‐lapse confocal images display the dynamics of Aβ‐accumulated mitochondria clearing. Images and quantification were achieved from living ARPE19 cells treated with FITC‐labeled Aβ (10 μM) and stained with MitoTracker (50 nM) after exposure to Aβ and HN. Colocalization of FITC‐labeled Aβ and MitoTracker Red was quantified as a percentage of the total MitoTracker Red area. Scale bar, 10 μm. (D) Dual‐color time‐lapse confocal images display the dynamic fusion process between mitochondria and lysosomes. Images and quantification were achieved from living ARPE19 cells stained with MitoTracker (50 nM) and LysoTracker (50 nM). Colocalization of MitoTracker Red with LysoTracker Green was quantified as a percentage of the total MitoTracker Red area. Scale bar, 10 μm. (E, F) Representative confocal images exhibit that bafiolomycin A1 (Baf.A1) counteracts the reduced level of colocalization between FITC‐labeled Aβ and MitoTracker Red induced by HN, while emphasizing the increased level of colocalization between MitoTracker Red and LAMP1 induced by HN. Colocalization of MitoTracker Red and FITC‐labeled Aβ or LAMP1 was quantified as a percentage of the total MitoTracker Red area. Scale bar, 10 μm. (G) Representative immunoblot and quantification of mitochondrial fractions show that HN enhances the translocation of LAMP1 to the mitochondria, resulting in a reduction in Aβ accumulation. Bafilomycin treatment further emphasizes the HN‐induced translocation of LAMP1 and the accumulation of Aβ within the mitochondria. Expression levels were normalized to the respective COX IV expression levels. (H) Representative immunoblot and quantification of the cytosolic fraction show that bafilomycin treatment effectively reveals HN‐induced LAMP1 depletion and Aβ accumulation. Expression levels were normalized to the respective GAPDH expression levels. The number of biologically independent experiments is 3 in (A‐D, G, H) and 5 in (E, F). Data are presented as mean ± SD. *p* values were determined using one‐way ANOVA with Tukey's multiple‐comparison analysis (A, B, E–H) and unpaired two‐tailed Student's *t*‐test (C, D). *p < 0.05, **p < 0.01, and ***p < 0.001.

Concurrently reducing intracellular Aβ levels, HN also decreased the abundance of p62 and increased the LC3II/I ratio, indicating enhanced autophagic processes. Aβ promoted the phosphorylation of Transcription factor EB (TFEB), leading to its retention in the cytosol. In contrast, HN reduced the phosphorylated (inactive) form of TFEB, accentuating its nuclear actions as the master regulator of lysosomal biogenesis, as evidenced by increased level of Lysosomal Associated Membrane Protein 1 (LAMP1) (Figure 2A) (Martina et al. 2014; Sardiello et al. 2009). Immunofluorescence analysis further showed that HN enhanced nuclear localization of TFEB in Aβ‐treated ARPE19 cells (Figure S1A,B). Additionally, HN significantly decreased the abundance of mitochondrial proteins such as TIM23, COX IV, and TOM20, suggesting the potential elimination of mitochondria through autophagy (Figure 2B). To determine whether Aβ‐accumulated mitochondria were undergoing elimination, live cell imaging showed the colocalization of mitochondria (Mitotracker) with FITC‐labeled Aβ or with lysosome (Lysotracker). In response to HN co‐treatment, the colocalization of FITC‐labeled Aβ with mitochondria decreased (Figure 2C), while the colocalization of lysosome with mitochondria increased (Figure 2D).

Next, we investigated whether HN enhances the lysosomal degradation of Aβ‐accumulated mitochondria by analyzing the colocalization of mitochondria with LAMP1 or FITC‐labeled Aβ. HN decreased the colocalization of mitochondria with Aβ and increased their colocalization with LAMP1 (Figure 2E,F). Bafilomycin A1 (Baf.A1) abolished HN‐induced removal of colocalized Aβ and mitochondria (Figure 2E). This suggests that HN‐induced Aβ removal might be mediated by autophago‐lysosomal degradation. Moreover, the colocalization between LAMP1 and mitochondria also notably increased with Baf.A1, indicating a potential accumulation of autophagosome‐engulfed mitochondria (Figure 2F).

Analysis of mitochondrial and cytoplasmic fractions revealed that HN reduced Aβ accumulation within mitochondria while promoting the translocation of LAMP1 from the cytosol to the mitochondrial fraction (Figure 2G,H). In the presence of Baf.A1, HN increased Aβ accumulation in both mitochondrial and cytosolic fractions, suggesting that HN enhances the clearance of Aβ through lysosomal degradation. Consistently, time‐dependent analysis revealed that HN transiently reduced mitochondrial mass‐related proteins, including TIM23, COX IV, and TOM20, at earlier time points, with restoration at 24 h (Figure S2A,B), indicating a dynamic and regulated turnover of mitochondria rather than sustained mitochondrial loss, supporting the notion that mitochondrial function is preserved.

### 2.3 HN Phosphorylates ULK1‐Beclin1 and Reverses Aβ‐Induced Mitochondrial Dysfunction via AMPK Activation

**FIGURE 3:**
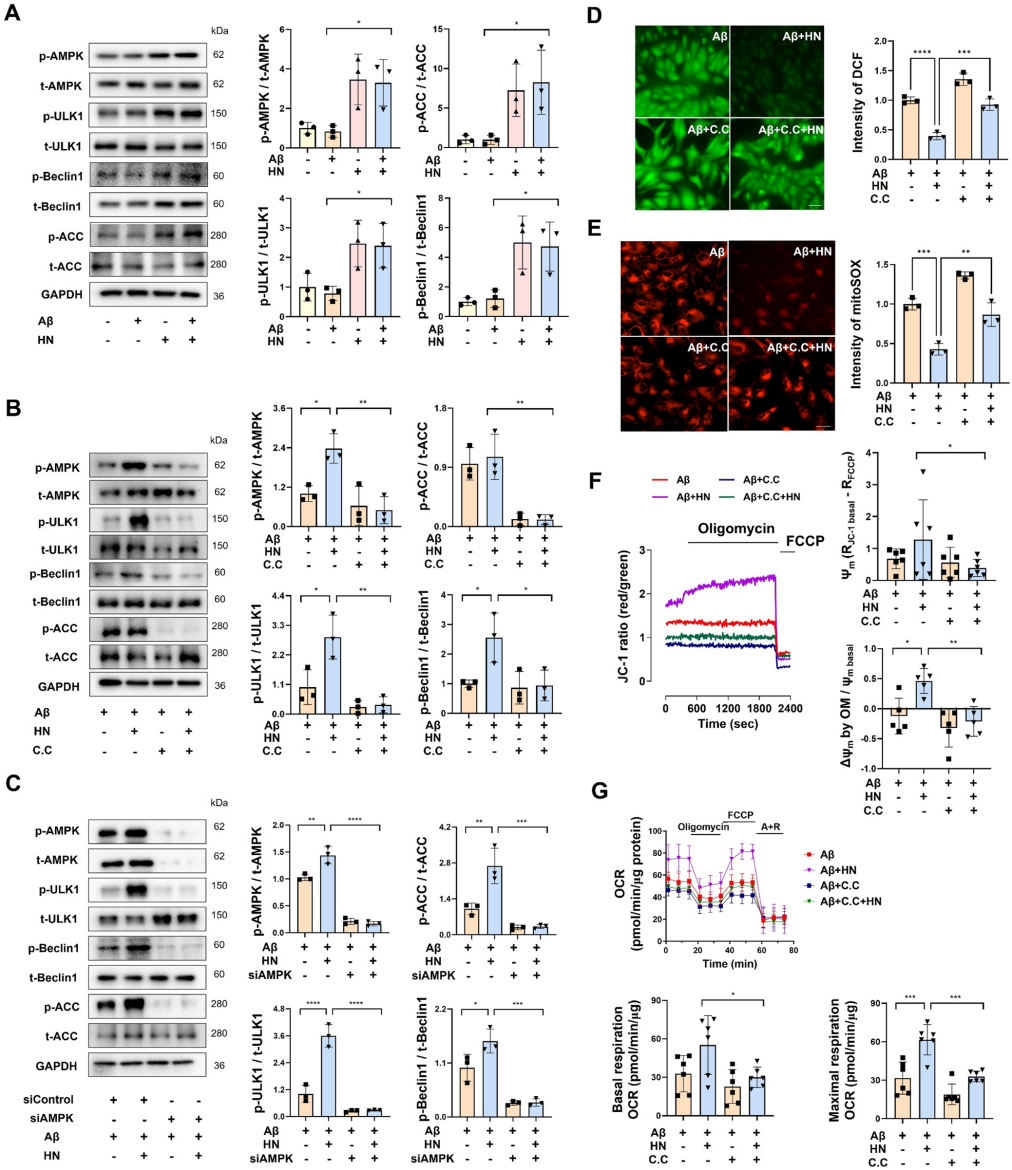
HN activates AMPK‐ULK1‐Beclin1 pathway and reverses Aβ‐induced mitochondrial dysfunction. (A–C) Representative immunoblots and quantification of total and phosphorylated protein levels of AMPK, ULK1, Beclin1, and ACC in ARPE‐19 cells. Effects of HN on the signaling enzymes' activation were evaluated under pre‐incubation with 10 μM Compound C (B) or knock‐down of AMPK by transfecting siRNA for 72 h (C). (D, E) DCF‐DA (5 mM) or mitoSOX (5 mM) was loaded and fluorescence images were analyzed for estimating cytosolic (D) or mitochondrial ROS (E) generation, respectively. (F, G) HN‐induced changes in mitochondrial membrane potentials (ΔΨm) (F) and oxygen consumption rate (OCR) (G) were measured under pre‐incubation with 10 μM Compound C. The number of biologically independent experiments is 6 in (G), 5 in (F), and 3 in (A–E). Data are presented as mean ± SD. *p* values were determined using one‐way ANOVA with Tukey's multiple‐comparison analysis. *p < 0.05, **p < 0.01, ***p < 0.001, and ****p < 0.0001. Scale bar, 20 μm.

We investigated whether HN affects AMPK‐ULK1 signaling related to the clearance of Aβ‐accumulated mitochondria. HN increases the phosphorylation levels of AMPK and its downstream target, acetyl‐CoA carboxylase (ACC), followed by phosphorylation of ULK1 and Beclin1 in ARPE19 cells (Figure 3A). These phosphorylations were entirely abrogated by preincubation with compound C (10 μM), an inhibitor of AMPK activation (Figure 3B). Genetic suppression of AMPK using RNA interference also substantially attenuated HN‐activated AMPK, ACC, ULK1, and Beclin1, demonstrating that HN induces AMPK‐dependent phosphorylations of ULK1 and Beclin1 (Figure 3C).

To further understand the dependency of HN's actions on AMPK, we examined the effect of pretreatment with compound C on Aβ‐induced ROS generation. As shown in Figure 3D,E, blocking AMPK activation with compound C effectively prevented Aβ‐triggered production of cytosolic ROS and mitochondrial superoxide. HN's abilities to increase the mitochondrial electrical gradient and to induce a hyperpolarizing response to oligomycin were blunted by co‐treatment with compound C (Figure 3F). Consistently, HN‐induced recovery of mitochondrial respiratory activities was abolished by compound C treatment (Figure 3G). These results suggest that HN induces AMPK‐dependent phosphorylation of ULK1 and Beclin1, which are responsible for the protective actions of HN against Aβ‐induced oxidative stress and mitochondrial dysfunction in the RPE.

### 2.4 HN Promotes Beclin1‐Parkin Interaction and Their Mitochondrial Translocation

**FIGURE 4:**
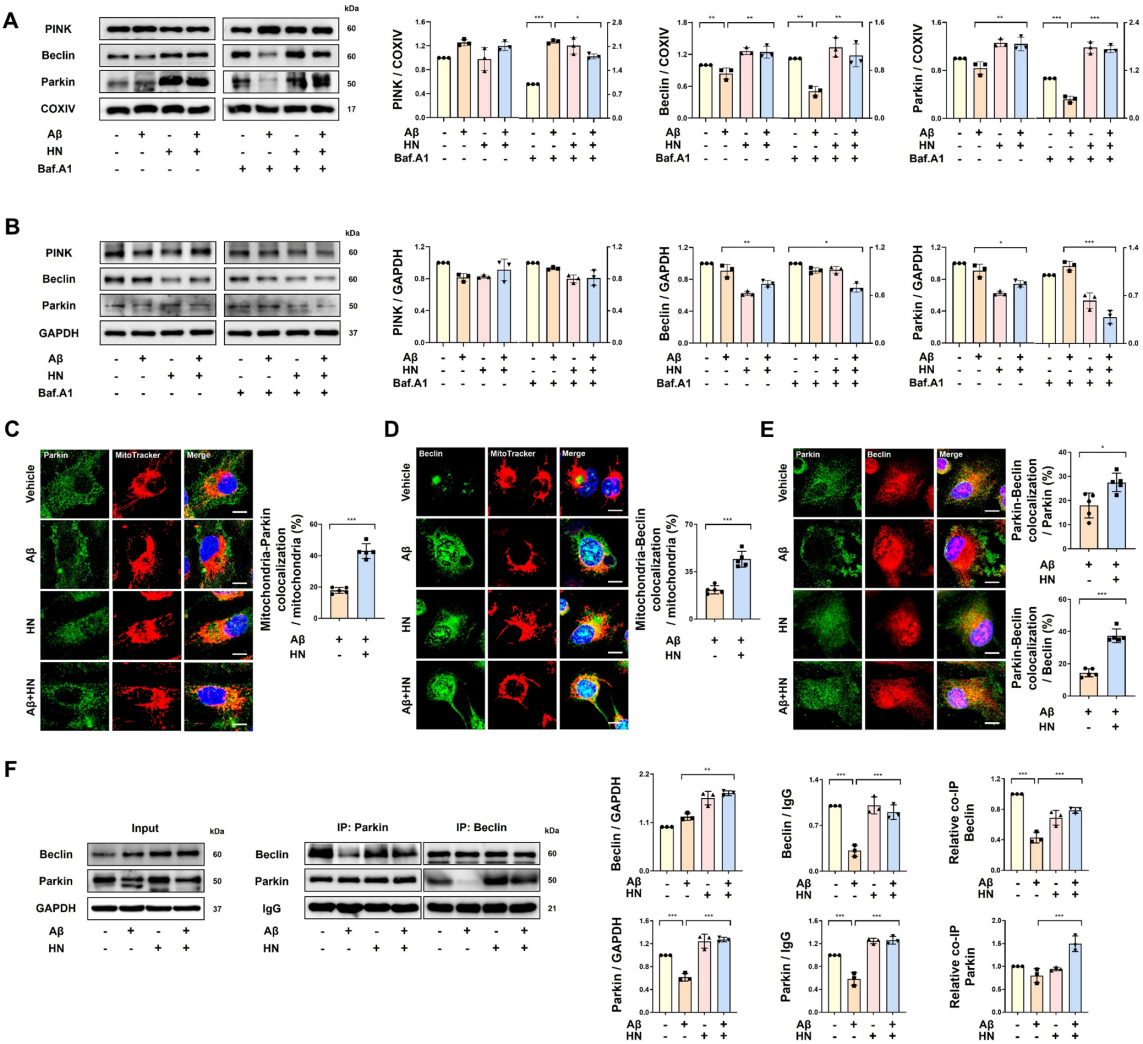
HN enhances the mitochondrial translocation of Parkin by promoting its interaction with Beclin1. (A) Representative immunoblot and quantification of mitochondrial fraction demonstrate that HN facilitates the translocation of both Parkin and Beclin1 to the mitochondria, leading to their accumulation in response to bafilomycin. Expression levels were normalized to the respective COX IV expression levels. (B) Representative immunoblot and quantification and of the cytosolic fraction show a decrease in Beclin1 levels in the cytoplasm due to promoted translocation to the mitochondria by HN. Bafilomycin enables the observation of Parkin translocation from the cytoplasm to the mitochondria, resulting in a decrease in the cytoplasmic level. Expression levels were normalized to the respective GAPDH expression levels. (C) Representative confocal images of MitoTracker Red and Parkin immunostaining depict that HN enhances the colocalization reduced by Aβ. The colocalization of MitoTracker Red with Parkin was quantified as a percentage of the total MitoTracker Red area. Scale bar, 10 μm. (D) Representative confocal images of MitoTracker Red and Beclin1 immunostaining depict that HN enhances the colocalization reduced by Aβ. The colocalization of MitoTracker Red and Parkin was quantified as a percentage of the total MitoTracker Red area. Scale bar, 10 μm. (E) Representative confocal images of Parkin and Beclin1 immunostaining depict that HN enhances the colocalization reduced by Aβ. The colocalization of Parkin with Beclin1 was quantified as a percentage of the total MitoTracker Red area. Scale bar, 10 μm. (F) Representative immunoblots and quantification show that HN enhances the interaction between Parkin and Beclin1, which is reduced by Aβ, as assessed by co‐IP using anti‐Parkin and anti‐Beclin1 antibodies. Input represents 10% of whole cell lysate from each sample as a control for protein expression. Quantification of input samples was normalized to GAPDH. Co‐immunoprecipitated proteins were quantified relative to IgG controls (Beclin1/IgG and Parkin/IgG), and the interaction between Parkin and Beclin1 was further expressed as relative co‐IP levels. The number of biologically independent experiments is 3 in (A, B, F) and 5 in (C‐E). Data are presented as mean ± SD. *p* values were determined using one‐way ANOVA with Tukey's multiple‐comparison analysis (A, B, F) and unpaired two‐tailed Student's *t*‐test (C–E). *p < 0.05, **p < 0.01, and ***p < 0.001.

We investigated whether HN‐induced phosphorylation of Beclin1 could facilitate the mitochondrial translocation of Parkin. To assess this, we analyzed the expression levels of Beclin1 and Parkin in mitochondrial and cytosolic fractions. As shown in Figure 4A,B, Aβ inhibited the mitochondrial translocation of both Beclin1 and Parkin, which was recovered by HN treatment. Imaging experiments confirmed that HN increased the mitochondrial localization of Parkin (Figure 4C) and Beclin1 (Figure 4D). Furthermore, the colocalization between Beclin1 and Parkin was also significantly increased by HN treatment (Figure 4E). Co‐immunoprecipitation studies revealed that Aβ suppressed the physical interaction between Beclin1 and Parkin, while HN restored their binding (Figure 4F).

### 2.5 HN Facilitates the Clearance of Aβ‐Accumulated Mitochondria by Promoting the Mitochondrial Translocation of Beclin1 and Parkin

**FIGURE 5:**
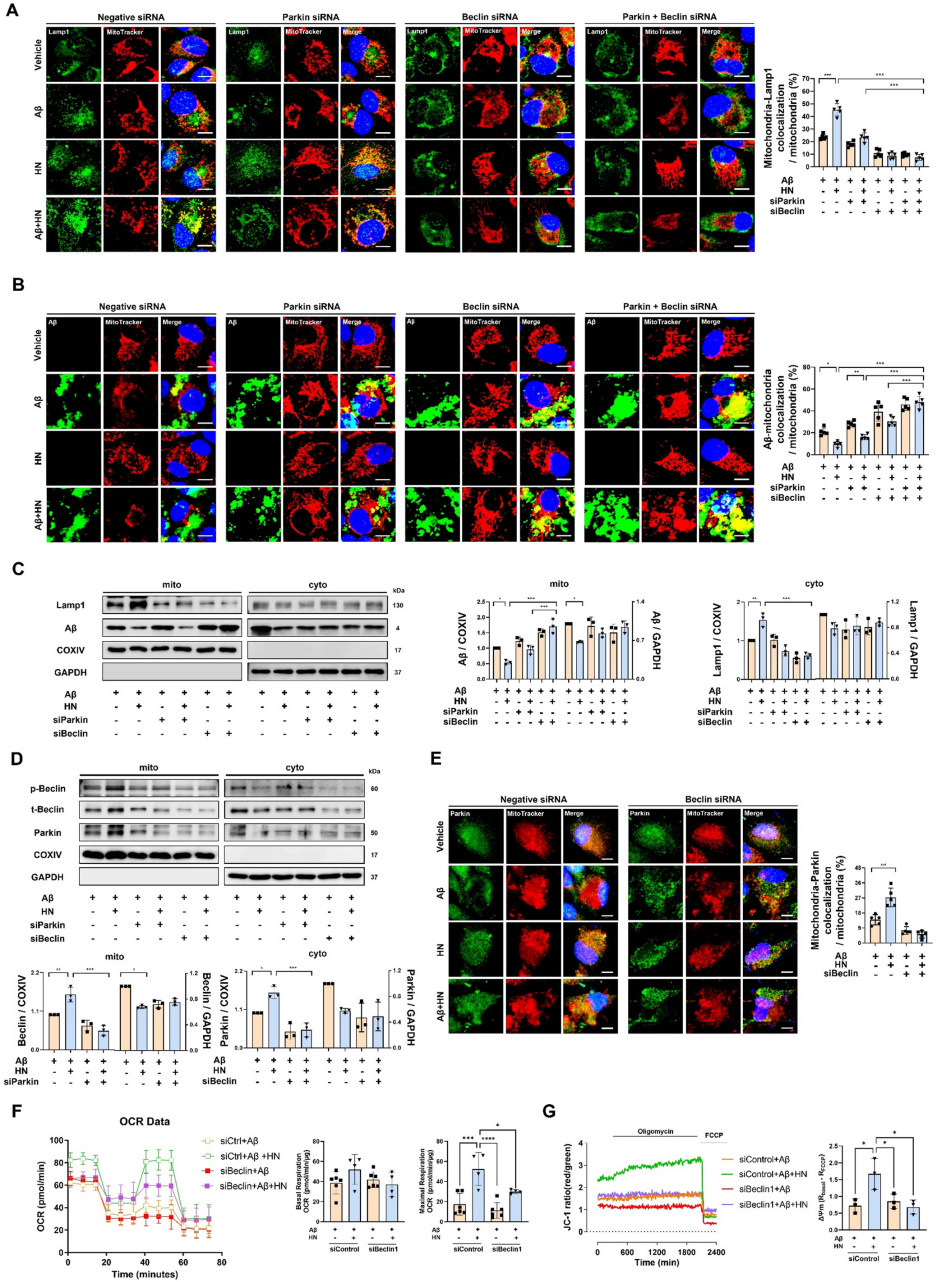
HN facilitates Parkin translocation to mitochondria upon Beclin1 dependency. (A) Representative confocal images show that colocalization of LAMP1 and MitoTracker induced by HN was reduced by the repression of Parkin or Beclin1 or both Parkin and Beclin1 simultaneously. Concurrent repression of Parkin and Beclin1 did not elicit a significant difference compared to the repression of Beclin1 alone. Scale bar, 10 μm. (B) Representative confocal images show that the decreased colocalization of FITC‐labeled Aβ and MitoTracker Red induced by HN was increased by the repression of Parkin or Beclin1 or both Parkin and Beclin1 simultaneously. Concurrent repression of Parkin and Beclin1 did not elicit a significant difference compared to the repression of Beclin1 alone. Scale bar, 10 μm. (C) Representative immunoblot and quantification of the mitochondrial and cytoplasmic fractions exhibit that HN's role in facilitating the recruitment of LAMP1 to mitochondria and the removal of Aβ‐accumulated mitochondria was significantly inhibited by Beclin1 repression rather than Parkin. (D) Representative immunoblot and quantification of the mitochondrial and cytoplasmic fractions exhibit that the role of HN in recruiting Parkin to mitochondria was significantly inhibited not only by Parkin repression but also notably by Beclin1 repression. Phosphorylated Beclin1 was additionally assessed in relation to mitochondrial localization. (E) Representative confocal images demonstrate that the HN‐enhanced colocalization of MitoTracker Red and Parkin is diminished in response to Beclin1 repression. Scale bar, 10 μm. (F) Oxygen consumption rate (OCR) analysis showing that HN increases mitochondrial respiratory capacity, which is attenuated by Beclin1 knockdown. Quantification of basal and maximal respiration is presented. (G) Mitochondrial membrane potentials (ΔΨm) assessed using JC‐1 staining demonstrates that HN‐induced recovery of ΔΨm is reduced in Beclin1‐deficient cells. The number of biologically independent experiments is 4–5 in (A, B, E, F) and 3 in (C, D, G). Data are presented as mean ± SD. *p* values were determined using one‐way ANOVA with Tukey's multiple‐comparison analysis. *p < 0.05, **p < 0.01, ***p < 0.001 and ****p < 0.0001.

We used siRNA to downregulate Parkin and Beclin1 and investigated their roles in the colocalization of LAMP1 or FITC‐labeled Aβ with mitochondria. Genetic suppression of Parkin using siRNA abolished HN‐induced augmentation of colocalization between LAMP1 and mitochondria. Similarly, Beclin1 knockdown reduced this colocalization, preventing HN‐stimulated mitochondria‐lysosome interaction. Time‐course analysis revealed that HN reduced Aβ‐mitochondria colocalization at 6 h, whereas no significant difference was observed at 24 h, suggesting progressive clearance of Aβ‐accumulated mitochondria at earlier time points (Figure S3A). LAMP1 recruitment to mitochondria appeared to remain elevated in cells treated with both Aβ and HN compared to Aβ alone throughout the time course, peaking at 6 h, reflecting continued lysosomal association with mitochondria (Figure S3B). Concurrent knockdown of Parkin and Beclin1 did not further inhibit colocalization beyond the effect of Beclin1 knockdown alone (Figure 5A). In addition, knockdown of Beclin1, either alone or in combination with Parkin, led to increased accumulation of Aβ in mitochondria, negating the action of HN (Figure 5B). However, knockdown of PINK1 did not affect HN‐induced mitochondria‐lysosome colocalization, LAMP1 recruitment to mitochondria, or the clearance of Aβ‐accumulated mitochondria (Figure S4A,B), indicating that HN‐mediated mitophagy is largely independent of the canonical PINK1 pathway. These findings suggest that HN's action on the degradation of Aβ‐accumulated mitochondria depends on both Parkin and Beclin1.

Immunoblot analysis for Aβ and LAMP1 in cytosolic and mitochondrial fractions, consistent with the immunostaining results, showed that HN‐induced removal of mitochondrial Aβ was decreased by Parkin downregulation, and further abolished by Beclin1 downregulation (Figure 5C). Moreover, downregulation of either Beclin1 or Parkin significantly hindered the mitochondrial translocation of the other protein (Figure 5D). Consistently, phosphorylated Beclin1 exhibited a similar pattern of change in relation to mitochondrial localization (Figure S5). Immunostaining results also demonstrated that mitochondrial localization of Parkin is markedly suppressed by Beclin1 knockdown, with no noticeable change observed in response to HN (Figure 5E). These results indicate that both Beclin1 and Parkin are required for their mitochondrial translocation and the recruitment of lysosome to mitochondria, leading to the removal of Aβ‐accumulated mitochondria. To further determine whether Beclin1‐dependent mitophagy contributes to mitochondrial functional recovery, we assessed mitochondrial functional parameters, including oxygen consumption rate and mitochondrial membrane potential (ΔΨm). HN treatment significantly increased maximal respiratory capacity, which was markedly attenuated upon Beclin1 knockdown. In addition, the recovery of mitochondrial membrane potential induced by HN was significantly reduced in Beclin1‐deficient cells. These findings provide functional evidence linking Beclin1‐dependent mitophagy to the restoration of mitochondrial function (Figure 5F,G).

### 2.6 HN Protects the Retina and RPE From Aβ‐Induced Degenerative Changes In Vivo

**FIGURE 6:**
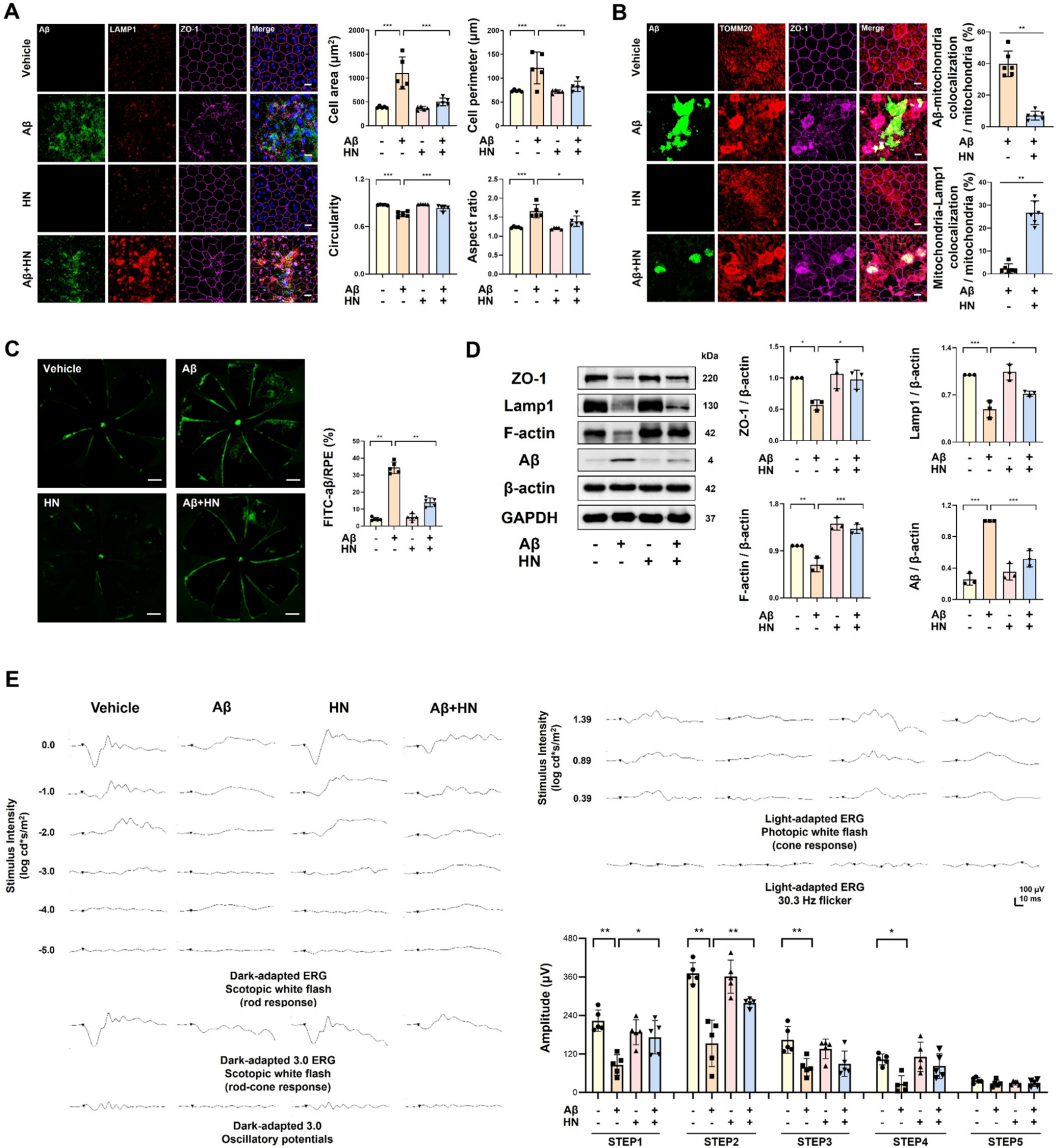
HN alleviates Aβ‐induced degenerative changes in the RPE and the retina. (A) Representative images of the RPE subretinally injected with FITC‐labeled Aβ demonstrate that HN restores the Aβ‐induced degenerative changes, characterized by increased cell size and irregularity. Morphological changes were quantified with cell size, perimeter, circularity, and aspect ratio. Scale bar, 10 μm. *n* = 6 mice. (B) Representative images of the RPE subretinally injected with FITC‐labeled Aβ, stained with TOM20 (red) and ZO‐1 (magenta), with quantification focusing on mitochondria in RCSs. Scale bar, 10 μm. *n* = 6 mice. (C) Localization and quantification of subretinally injected FITC‐labeled Aβ in flat‐mounted RCSs. HN reduces the distribution area of Aβ in the entire RCSs. Scale bar, 500 μm. *n* = 5 mice. (D) Representative immunoblot and densitometry graphs of the RPE and choroid lysate demonstrate promoted Aβ clearance and alleviation of tight junction disruption. Three biologically independent experiments were performed. (E) Representative scotopic and photopic full‐field ERG waveforms illustrating Aβ‐induced amplitude decrease, partially rescued by HN. *n* = 5 mice. Data are presented as mean ± SD. *p* values were determined using one‐way ANOVA with Tukey's multiple‐comparison analysis (A, D, E, F) and unpaired two‐tailed Student's *t*‐test (B, C). *p < 0.05, **p < 0.01, and ***p < 0.001.

To assess the role of HN in modulating Aβ accumulation and distribution, FITC‐labeled Aβ1‐42 (10 μM) was subretinally injected into mice. Three days later, the RPE exhibited degenerative changes, losing its characteristic cobblestone‐like morphology and disruption of ZO‐1, accompanied by intracellular localization of FITC‐labeled Aβ (Figure S6). Quantitative morphological analysis showed enlarged and irregular cells. Notably, Aβ also induced nuclear enlargement and multinucleation, consistent with the observations in primary marmoset RPE cells. Intravitreal co‐administration of HN effectively inhibited the Aβ‐induced disruption of the external blood‐retinal barrier, significantly restoring normal RPE morphology (Figure 6A). HN reduced the colocalization of Aβ with TOM20 while enhancing the colocalization of TOM20 and LAMP1 (Figure 6B), suggesting that HN enhances the removal of Aβ‐accumulated mitochondria.

The overall deposition of FITC‐labeled Aβ throughout the whole RPE was reduced by HN treatment (Figure 6C). Additionally, HN downregulated Aβ levels and upregulated LAMP1 expression across the RPE, preventing the decrease in ZO‐1 and F‐actin expression (Figure 6D). Electroretinography (ERG) measurements taken 3 days after subretinal Aβ injection showed subnormal amplitudes in both dark‐adapted and light‐adapted retinas. HN co‐treatment substantially improved these retinal responses, particularly under dark‐adapted conditions, improving retinal function (Figure 6E).

## 3 Discussion

Our results demonstrated that oligomeric Aβ1‐42 entered both ARPE19 cells and primary marmoset RPE cells, inducing oxidative stress, ZO‐1 disruption, and impairment of barrier integrity. Although ARPE19 cells are widely used in ocular research, they do not fully recapitulate the native RPE phenotype, particularly in terms of gene expression, barrier function, and cellular heterogeneity (Markert et al. 2022). To complement this model, we employed primary marmoset RPE cells, which more closely resemble in vivo RPE characteristics and were utilized for morphological and barrier integrity assessments following prolonged differentiation under serum‐free conditions with B27 supplementation (Jang et al. 2023). Subretinal injection of Aβ provoked several features indicative of damage in both the RPE and the retina. Significant degenerative changes, including disruption of the characteristic polygonal morphology and multinucleation, were observed at 3 days post‐injection, similar to previous reports (Liu et al. 2015). Additionally, both dark‐adapted and light‐adapted responses in the retina were diminished. HN, as an autophagy inducer, improved muscle functions in aged mice and extended lifespan in HN transgenic worms (Kim et al. 2022). While HN has been linked to autophagy and CMA, our findings suggest that its role extends to mitophagy, particularly through the recruitment of Beclin1 and Parkin to damaged mitochondria in the RPE. In this study, Aβ‐induced degenerative changes and oxidative stress were alleviated by HN in the RPE. HN also regulated several key factors involved in autophagosome‐lysosome formation, such as p62, LC3, LAMP1, and TFEB phosphorylation, facilitating the removal of intracellular Aβ aggregates. Notably, HN reduced overall mitochondrial mass and decreased Aβ‐accumulated mitochondria, suggesting that HN‐mediated activation of the autophagy‐lysosomal pathway alleviated mitochondrial dysfunction by eliminating damaged mitochondria.

Mitophagy is essential for RPE homeostasis, and its impairment has been increasingly implicated in AMD pathogenesis, particularly in the context of mitochondrial dysfunction and aging (Datta et al. 2022; Fisher et al. 2022; Tong et al. 2022). In AD, Aβ accumulates within mitochondria and disrupts mitochondrial function (Caspersen et al. 2005; Chen and Yan 2007; Pinho et al. 2014; Sorrentino et al. 2017). Given the shared pathological features between AD and AMD, including extracellular deposition (Ding et al. 2008; Mody and Joshi 2023), we extended this approach to the RPE with potential therapeutic relevance. In our study, Aβ was observed to localize to mitochondria in RPE cells, HN treatment was associated with reduced Aβ‐mitochondria colocalization and enhanced lysosomal involvement. This effect was further supported by dynamic colocalization patterns that alternated between appearance and disappearance upon HN treatment, contrasting with the static colocalization state observed between Aβ and mitochondria in live‐cell time‐course analysis. HN also increased the delivery of mitochondria to lysosomes compared to Aβ treatment alone. By inhibiting autophagic flux using bafilomycin, which prevents autophagosome acidification and autophagosome‐lysosome fusion, we observed cumulative intramitochondrial trafficking of Aβ and LAMP1 upon HN treatment, indicating that HN actively enhanced the import of Aβ into mitochondria and facilitated the removal of Aβ‐accumulated mitochondria. These findings suggest that mitochondrial uptake of Aβ might play a role in the RPE degenerative changes beyond oxidative stress, highlighting the potential of clearing damaged mitochondria as a crucial strategy to treat age‐related diseases like AMD.

Parkin plays crucial roles in activating mitophagy and promotes autophagosome maturation through interaction with Beclin1, a process involved in the autophagic clearance of Aβ (Lonskaya et al. 2013; Nixon and Yang 2011). Our findings demonstrate that, in the absence of Beclin1, the recruitment of Parkin to mitochondria is suppressed. Similarly, Beclin1 recruitment to mitochondria is reduced without Parkin, indicating that these proteins are mutually dependent on each other for their mitochondrial translocation. HN significantly enhances the interaction between Parkin and Beclin1, restoring their translocation to mitochondria. Even in the absence of Parkin, HN can still facilitate the clearance of Aβ‐accumulated mitochondria, whereas this clearance is markedly compromised upon depletion of Beclin1. Simultaneous depletion of Parkin and Beclin1 does not exhibit discernible differences compared to depletion of Beclin1 alone, suggesting that Parkin‐mediated mitophagy by HN is exclusively dependent on Beclin1. Additionally, the enhancement of LAMP1 delivery to mitochondria induced by HN is diminished in the absence of Beclin1, either alone or in combination with Parkin. The recruitment of Parkin to mitochondria by HN is also deficient when Beclin1 is depleted. Reduction of PINK1, either alone or with Parkin, did not significantly impede the HN‐facilitated clearance of Aβ‐accumulated mitochondria or LAMP1 delivery, indicating that HN activates mitophagy through a non‐canonical, Beclin1‐Parkin axis rather than the classical PINK1‐dependent pathway.

The sequential phosphorylation and activation of the AMPK‐ULK1‐Beclin1 pathway are recognized as crucial in autophagy induction and regulation (Kim et al. 2011; Laker et al. 2017; Menon and Dhamija 2018; Russell et al. 2013). ULK‐mediated phosphorylation of UVRAG‐bound Beclin1 also contributes to autophagosome maturation. Compound C, an ATP‐competitive inhibitor of AMPK, hindered HN‐induced AMPK, ULK1, Beclin1, and ACC phosphorylation, suggesting that HN promotes Parkin‐mediated activation for the clearance of Aβ‐accumulated mitochondria through AMPK‐triggered phosphorylation of ULK1 and Beclin1. Humanin significantly increased AMPK phosphorylation and the downstream phosphorylation of ACC, ULK1, and Beclin1 in our study. However, the upstream mechanism responsible for AMPK activation was not directly investigated. It is possible that Humanin activates AMPK through multiple non‐mutually exclusive pathways, including receptor‐mediated signaling, modulation of intracellular metabolites, alterations in cellular redox or energy status, or mitochondrial/metabolic stress–dependent signaling. Further studies will be required to delineate the precise upstream mechanisms underlying Humanin‐induced AMPK activation.

In summary, HN effectively inhibits Aβ‐induced RPE degenerative changes by enhancing the clearance of Aβ‐accumulated mitochondria, and facilitating the recruitment of Beclin1 and Parkin to mitochondria through the upregulation of the AMPK‐ULK1‐Beclin1 signaling pathway. Beclin1 activation via AMPK was required for Parkin translocation to mitochondria, underscoring its pivotal role in HN‐induced mitochondrial quality control. The present study employed an acute subretinal Aβ injection model with a short observation period of 3 days, which does not fully recapitulate the chronic and progressive nature of dry AMD; therefore, future studies using chronic AMD models with extended follow‐up periods are required to validate the long‐term therapeutic potential of HN. Nevertheless, our findings highlight HN as a promising therapeutic candidate for AMD.

## 4 Materials and Methods

### 4.1 Animals

All animal procedures were approved by the Institutional Animal Care and Use Ethics Committee of Seoul National University (IACUC No. 231103–3), and all efforts were made to minimize animal suffering and reduce the number of animals used. Six‐week‐old male C57BL/6J mice (18–22 g, Orient Bio Inc., Sungnam, Korea) were housed in a pathogen‐free barrier environment throughout the study. The animals were anesthetized by intraperitoneal injection of a mixture of Zoletil 50 (50 mg/kg) and xylazine (5 mg/kg). Before the injection, the animals received tropicamide (Tropherin) eye drops for dilation. A step incision in the pars plana region of the sclera was made using a 26G needle, and subretinal (SR) and intravitreal (IVT) injections were carried out using a Hamilton syringe (Hamilton, Bonaduz, Switzerland) under a surgical microscope (Leica Microsystems Ltd., Wetzlar, Germany). The animals received an SR injection of 1 μL (10 μM) of oligomeric FITC‐labeled Aβ1‐42 (#50–194‐6621, Bachem) and an IVT injection of 1 μL of HN (20 μM) or PBS.

### 4.2 Immunofluorescence

Three days after the injection, the animals were sacrificed, and their eyes were fixed by immersion in 4% paraformaldehyde for 15 min at room temperature. The RPE‐choroid‐scleral complexes (RCSs) were carefully separated from the eyeball using a dissecting microscope (Nikon SM2745T, Nikon Corporation, Japan) and incubated with Perm/Block solution (0.2% Triton‐X 100 and 0.3% BSA in PBS). Subsequently, they were incubated with primary antibodies in the same solution overnight at 4°C, followed by incubation with secondary antibodies for 1 h at room temperature in a dark room. Nuclei were counterstained with 1 μg/mL 4′,6′‐diamidino‐2‐phenylindole (DAPI, Sigma Aldrich) for 5 min at room temperature. After washing with PBS, the RCSs were mounted using Fluoromount Aqueous Mounting Medium (DAKO, Glostrup, Denmark) and observed under a confocal microscope (Leica TCS STED, Leica Microsystems Ltd., Wetzlar, Germany). For ARPE19 cells and primary marmoset RPE cells, fixation, permeabilization, blocking, and antibody incubation were performed as described above. All primary, conjugated, and secondary antibodies used in this study are listed in Table S1.

### 4.3 Image Analysis

Six z‐stack images were taken in a clockwise direction from between the central and equatorial regions within a single RCS, as well as from differentiated ARPE19 cells and primary marmoset RPE cells, as previously described (Kim et al. 2021). For morphological analysis of individual cells in each image, we segmented RPE cells by extracting the image that captured cell borders stained with ZO‐1. Using a machine‐learning‐based ImageJ plugin (National Institutes of Health, Bethesda, MD, USA), Trainable Weka Segmentation, the image was binarized. Following a sequence of steps including despeckling, skeletonization, and removal of abnormally segmented cells, including those at the cut edge, we performed several morphometric analyses on the skeletonized image with thin cell borders. We calculated the morphological characteristics of each cell, including cell area, perimeter, aspect ratio (major axis length/minor axis length), and circularity ([4 × area × *π*]/perimeter (Biscetti et al. 2017)).

Colocalization analysis was performed on images after deconvolution and thresholding using ImageJ software (National Institutes of Health, Bethesda, MD, USA) on a minimum of 20 cells per slide and a total of at least 50 cells from 3 to 5 biologically independent experiments. Thresholding was applied to each image, and the adequacy of the threshold was manually validated by comparing with the original image. The same threshold value was applied to all images within the same experiment. The particle number was quantified using the ’analyze particles’ function in thresholded single sections with the size (pixel^2^) setting from 0.1 to 10 and circularity from 0 to 1. Cellular fluorescence intensity was expressed as the mean integrated density as a function of individual cell size. The percentage of colocalization was calculated in single z‐stack sections of deconvolved images (Turco et al. 2021).

### 4.4 Immunoprecipitation

Protein samples from cell lysates were precleared using 50 μL of packed protein A‐crosslinked 4% beaded agarose (Cell Signaling Technology, Beverly, MA, USA) at 4°C for 1 h. The beads were removed using centrifugation, and the supernatant was collected. The supernatant was incubated with Parkin (#39–0900, Invitrogen, 1:50), Beclin1 (#PA5‐96649, Invitrogen, 1:50), and PINK1 (#6946, Cell Signaling Technology, 1:50) antibodies at 4°C with constant mixing for 12 h. The immune complex was captured following the addition of packed agarose protein A beads (50 μL/500 μL supernatant) for 2 h at 4°C. Bead separation was achieved using centrifugation (13,000 × *g* for 25 min), and the supernatant was removed. Subsequently, the samples were subjected to immunoblot analysis with primary and appropriate secondary antibodies. The specificity of immunoprecipitation was confirmed using negative control reactions performed with a rabbit IgG or mouse IgG control without a primary antibody. The primary and secondary antibodies used in this study are listed in Table S1.

### 4.5 Immunoblot Analysis

RCSs and ARPE19 cells were lysed in RIPA buffer (1% Triton X‐100) containing the protease inhibitor (GenDEPOT) and the phosphatase inhibitor (GenDEPOT). Additionally, cells were fractionated into mitochondrial and cytoplasmic components using a mitochondrial/cytoplasmic fractionation kit (ab65320, Abcam), according to the manufacturer's instructions. Briefly, cells were homogenized using a pestle homogenizer and then centrifuged at 700 × *g* for 10 min. The supernatant was re‐centrifuged at 10,000 × *g* for 30 min to collect the supernatant (cytoplasmic fraction) and pellet (mitochondrial fraction). The purity of the cytosolic and mitochondrial fractions was confirmed by immunoblot analysis using anti‐GAPDH and anti‐COX IV antibodies, respectively. Protein content was measured using the BCA protein assay kit (Pierce, Rockford, IL, USA). Proteins were separated using SDS‐PAGE and transferred onto polyvinylidene fluoride (PVDF) membranes (Merck Millipore Ltd., Tullagreen, Ireland). The PVDF membrane was blocked using non‐fat milk and incubated with TBST, followed by sequential incubation with primary and appropriate secondary antibodies. Signals were visualized with SuperSignal West Pico Chemiluminescent Substrate (Thermo Fisher Scientific). Immunoreactive bands were visualized using ImageQuantTMLAS4000 (GE Healthcare, USA) according to the manufacturer's instructions. Densitometric analysis was performed using ImageJ software, and the results were normalized to those of GAPDH. The primary and secondary antibodies used in this study are listed in Table S1.

### 4.6 Cell Culture and Drug Treatment

The ARPE19 cell line obtained from the American Type Culture Collection (ATCC, Manassas, VA, USA) was cultured in a 1:1 (vol/vol) mixture of Dulbecco's Modified Eagle Medium (DMEM) and Ham's F‐12 (Welgene, Daegu, Korea) containing 10% FBS (Gibco, Waltham, MA, USA) and 1% penicillin/streptomycin (Gibco). Primary RPE cells were isolated from the common marmoset ( *Callithrix jacchus* ), a small New World monkey, at the Marmoset Model Network Center in Seoul National University Hospital (IACUC No. 22‐0069), as previously described (Jang et al. 2023). Cells were cultured in DMEM supplemented with N1 supplement (1%; Sigma Aldrich), penicillin/streptomycin (1%; Gibco), nonessential amino acids (1%; Sigma Aldrich), taurine (0.25 g/L; Sigma Aldrich), hydrocortisone (20 μg/L; Sigma Aldrich), triiodothyronine (13 ng/L; Sigma Aldrich), and 10% FBS (Gibco). Both ARPE19 cells and primary marmoset RPE cells were cultured on Transwell membranes with 0.4 μm pores (Corning Costar) to facilitate differentiation for morphological analysis and TEER measurement. ARPE19 cells were maintained in DMEM containing 1% FBS for 3 months (Ahmado et al. 2011), while primary marmoset RPE cells were cultured in serum‐free medium supplemented with B27 (Gibco) for at least 4 weeks (Jang et al. 2023). Cells grown on Transwell permeable supports were maintained with 200 μL of media in the apical chamber and 700 μL in the basal chamber. Human induced pluripotent stem cells (iPSCs) were generated from human fibroblasts (ATCC CRL‐2097) by reprogramming with plasmid vectors encoding OCT4, SOX2, KLF4, and c‐MYC, as previously described (Kim et al. 2017). iPSC‐derived RPE cells were generated using a previously described differentiation protocol (Surendran et al. 2022). Cells were maintained at 37°C and 5% CO_2_ and were regularly tested for mycoplasma.

Oligomeric Aβ1‐42 (# 50‐194‐6585, Bachem, 10 μM) with or without HN (20 μM) was added at the time points for immunoblot analysis and immunofluorescence. To assess the colocalization of mitochondria and Aβ by immunostaining, cells were treated with FITC‐labeled Aβ1‐42 (#50‐194‐6621, Bachem, 10 μM) with or without HN (20 μM). Bafilomycin A1 (10 nM; Sigma Aldrich) was used as a lysosomal function by selectively inhibiting vacuolar‐type H^+^‐ATPase (V‐ATPase), thereby preventing lysosomal acidification and blocking lysosomal degradation.

### 4.7 RNA Interference

ARPE19 cells were transfected with Silencer Select siRNA targeting Parkin (ID: s502575; Invitrogen) or Beclin1 (IDL 137198; Invitrogen) using the Lipofectamine RNAiMAX reagent (Invitrogen) when the cells reached 60%–80% confluence, according to the manufacturer's instructions. Silencer Select Negative Control No. 1 siRNA (Invitrogen) served as the negative control. Briefly, 3 μL of RNAiMAX transfection reagent was mixed with 50 μL of Opti‐MEM medium (Thermo Fisher Scientific). Then 1 μL of siRNA (10 μM) was diluted with 50 μL of Opti‐MEM medium. The diluted siRNA was mixed with transfection reagent at room temperature for 5 min, and then added to ARPE19 cells. Following 24 h of transfection, cells were treated with Aβ (10 μM) with or without HN (20 μM).

### 4.8 ERG Analysis

Mice were anesthetized with an intraperitoneal injection of a mixture of Zoletil 50 (2.25 mg/kg) and xylazine (0.7 mg/kg) after dark adaptation for 16 h. Pupils were dilated with an eye drop of 0.5% tropicamide. Full‐field ERGs were recorded using the universal testing and electrophysiologic system 200 (UTAS E‐2000, LKC Technologies, Gaithersburg, MD, USA), as previously described (Gresh et al. 2003). The responses were recorded at 2 K gain, using a 69 Hz notch filter and bandpass filtering between 0.1 and 1500 Hz. In the light‐adapted state (photopic), cone responses were isolated and recorded in response to a single flash at 0 dB and a flicker sequence of 30 Hz with 30 cd/m^2^ background illumination to desensitize the rods. A‐wave amplitude was measured from baseline to the lowest negative voltage, while peak b‐wave amplitude was measured from the lowest point of the a‐wave to the highest peak of the positive b‐wave.

### 4.9 TEER Measurement

TEER measurements were taken using an EVOM3 epithelial volt‐ohmmeter and STX4 electrode (World Precision Instruments, Sarasota, FL, USA) according to the manufacturer's instructions. The electrodes were sterilized with 70% ethanol, rinsed in ddH_2_O, and equilibrated in pre‐warmed culture medium. To minimize the influence of temperature, measurements were performed in the hood within 5 min after the cells were taken out of the incubator. Measurements were repeated in five independent experiments, with at least three separate wells recorded per experiment to obtain an average value. Net TEER (Ω·cm^2^) was calculated by subtracting the value of a blank insert from the experimental value and multiplying it by the area of the insert membrane (0.33 cm^2^ for 25‐well insert).

### 4.10 Live Imaging

For live‐cell imaging, ARPE19 cells were seeded in glass bottom dishes (Nunc, Thermo Fisher Scientific) at a density of 5 × 10^4^ cells per dish in a growth medium. After an overnight incubation, cells were treated with Aβ (10 μM) with or without HN (20 μM) in DMEM/F‐12 without phenol red (Welgene) containing 10% FBS (Gibco) and 1% penicillin/streptomycin (Gibco). Cells were stained with LysoTracker Green (#8783, Cell Signaling Technology, 50 nM) and MitoTracker Red (#9082, Cell Signaling Technology, 50 nM) for 30 min in a humidified CO_2_ incubator and observed under a confocal microscope (Leica STED, Leica Microscope Ltd., Wetzlar, Germany) for 1 h. *Z*‐stacks ranging from 80 to 100 μM were taken with a step size of 4–5 μM at intervals of 3 min. Temperature and CO_2_ levels were controlled with a stage‐top incubator.

### 4.11 Measurement of ROS

To measure intracellular reactive oxygen species (ROS) production, ARPE‐19 cells were incubated with chloromethyl‐H2‐dichlorofluorescein diacetate (CM‐H2DCF‐DA), a cell‐permeable, non‐fluorescent probe. Upon entering the cell, intracellular esterases cleave the diacetate groups, and the chloromethyl group facilitates intracellular retention via binding to glutathione and thiol‐containing proteins. The deacetylated probe is then rapidly oxidized by intracellular ROS to produce the highly fluorescent 2,7‐dichlorofluorescein (DCF), detected at excitation/emission wavelengths of 485/535 nm. Briefly, cultured ARPE‐19 cells, treated with or without TGF‐β1 and specific inhibitors as specified, were exposed to a 5 μM concentration of CM‐H2DCF‐DA for 20 min at 37°C. Following incubation, the cells were washed with Krebs‐Ringer bicarbonate (KRB) solution (135 mM NaCl, 3.6 mM KCl, 2 mM NaHCO3, 0.5 mM NaH2PO4, 0.5 mM MgSO4, 1.5 mM CaCl2, and 10 mM HEPES; pH 7.4). Fluorescence images (excitation/emission: 490/535 nm) were captured using an IX81 fluorescence microscope (Olympus, Tokyo, Japan) with a confocal spinning disk, and fluorescence intensity was quantified using Metamorph 6.1 software (Molecular Devices, Sunnyvale, CA). for the selective detection of mitochondrial superoxide, ARPE‐19 cells were treated with MitoSOX Red mitochondrial superoxide indicator. MitoSOX reagent was prepared to a 5 μM concentration in KRB solution and used to treat ARPE‐19 cells for 15 min at 37°C. Fluorescence intensity was then measured by confocal microscopy and analyzed using MetaMorph software.

### 4.12 Mitochondrial Oxygen Consumption Rate Measurement

An Extracellular Flux Analyzer XF‐96 (Seahorse Bioscience, Agilent, Santa Clara. CA, USA) was used to determine cellular oxygen consumption rate (OCR) in the cells XF Base Media was used as an assay medium including glucose 22 mM, pyruvate I mM and glutamine 2 mM, which was used to incubate with cells for 45–60 min before basal measurement. After 20 min of measurement for the baseline, the effect of oligomycin (2uM), an ATP synthase inhibitor that blocks mitochondrial ATP production, FCCP (2 μM), rotenone/antimycin A (0.5 μM/1.5 μM) were recorded during OCR measurement.

### 4.13 Mitochondrial Membrane Potential Measurement

Mitochondrial membrane potentials (Δψm) were measured using 5,5′,6,6′‐tetrachloro‐1,1′,3,3′‐tetraethylbenzimidazolylcarbocyanine iodide (JC‐1, catalog no. T3168, Molecular Probes, Thermo Fisher Scientific), a lipophilic cationic dye. Due to its positive charge and lipophilic nature, JC‐1 monomers accumulate in mitochondria where the membrane potential is highly negative (approximately −150 to −200 mV), causing them to aggregate into polymers. JC‐1 monomers emit green fluorescence (excitation at 490 nm, emission at 535 nm), while the polymers emit red fluorescence (excitation at 540 nm, emission at 590 nm). The fluorescence signals were recorded using a fluorescence microplate reader (FlexStation III, Molecular Devices). The Δψm was estimated by calculating the ratio of red to green fluorescence (590 nm/535 nm).

### 4.14 Statistical Analysis

The results are presented as the mean ± SD. One‐way ANOVA followed by Tukey's post hoc test was used for comparisons among three or more groups, and unpaired two‐tailed Student's *t*‐test was used for comparisons between two groups. No statistical methods were used to predetermine the sample size. Mice were randomly allocated to experimental groups. No blinding method was used for SR and IVT injection. There were no animal exclusion criteria. The variance was similar between groups that were statistically compared. One‐way ANOVA and Student's *t*‐test analysis were performed using GraphPad Prism (San Diego, CA, USA) version 10.0 software. Data was considered statistically significant at *p* < 0.05.

## Author Contributions

Conceptualization was carried by Kyu‐Sang Park and Jeong Hun Kim. Investigation was performed by Ha Young Jang, Suyeon Choi and Sooyun Kim. Data analysis and visualization were performed by Ha Young Jang, Suyeon Choi, Soo‐Jin Kim, Tae Geol Lee, Kyu‐Sang Park, and Jeong Hun Kim. Writing of the original draft was carried out by Ha Young Jang and Kyu‐Sang Park. Writing – review and editing of the text was performed by Ha Young Jang, Kyu‐Sang Park, Dong Hyun Jo, and Jeong Hun Kim.

## Funding

This work was supported by the National Research Foundation of Korea (2022M3A9E4017127, RS‐2023‐00260351). Kun‐hee Lee Child Cancer and Rare Disease Project (202200004004). Seoul National University Hospital (18‐2023‐0010). National Research Council of Science and Technology (GTL25021‐000).

## Ethics Statement

The authors have nothing to report.

## Conflicts of Interest

The authors declare no conflicts of interest.

## Supporting information


**Figure S1:** HN enhances nuclear localization of TFEB in Aβ‐treated iPSC‐derived RPE cells. (A) Representative confocal images and quantification of iPSC‐derived RPE cells showing TFEB (green), ZO‐1 (red), and nuclei (DAPI, blue) following treatment with Aβ alone or in combination with HN. Aβ treatment resulted in reduced nuclear localization of TFEB, whereas co‐treatment with HN increased TFEB nuclear localization. (B) Quantification of nuclear TFEB signal. Scale bar, 10 μm. *p < 0.05, **p < 0.01, and ***p < 0.001. Data are mean ± S.D. from biologically independent experiments (*n* = 3).
**Figure S2:** Time‐dependent changes in mitochondrial mass‐related protein expression following Aβ and HN treatment. (A) Representative immunoblot images showing the expression of mitochondrial mass‐related proteins TIM23, COX IV, and TOM20 in ARPE19 cells treated with Aβ alone or in combination with HN at 3, 6, and 24 h post‐treatment. The 6 h time point corresponds to the data presented in Figure 2B. (B) Densitometric quantification of band intensities for TIM23, COX IV, and TOM20 at each time point, normalized to GAPDH. *p < 0.05, **p < 0.01, and ***p < 0.001. Data are mean ± S.D. from biologically independent experiments (*n* = 3).
**Figure S3:** Dynamics in colocalization of mitochondria with Aβ and LAMP1 by HN throughout the time course. (A) Representative confocal images and quantification of ARPE19 cells treated with FITC‐labeled Aβ alone or with HN at 3, 6, and 24 h after treatment, tracking the colocalization of FITC‐labeled Aβ and mitochondria. (B) Representative confocal images and quantification of ARPE19 cells treated with Aβ alone or with HN at 3, 6, and 24 h after treatment, tracking the colocalization of LAMP1 and mitochondria. Scale bar, 10 μm. *p* values were determined using one‐way ANOVA with Tukey's multiple‐comparison test (A, B). *p < 0.05 and **p < 0.01, and ***p < 0.001. Data are mean ± S.D. from biologically independent experiments (*n* = 5).
**Figure S4:** HN is not involved in PINK‐mediated recruitment of LAMP1 to mitochondria and clearance of Aβ‐accumulated mitochondria. (A) Representative confocal images and quantification of ARPE19 cells to assess the role of PINK in the recruitment of LAMP1 to mitochondria by HN. (B) Representative confocal images and quantification of ARPE19 cells to evaluate the role of PINK in the clearance of Aβ‐accumulated mitochondria by HN. *p* values were determined using one‐way ANOVA with Tukey's multiple‐comparison test (A, B). *p < 0.05 and **p < 0.01, and ***p < 0.001. Data are mean ± S.D. from biologically independent experiments (*n* = 3).
**Figure S5:** Quantification of phosphorylated Beclin1 and total Beclin1 distribution between mitochondrial and cytosolic fractions. (A) Quantification of phosphorylated Beclin1 (p‐Beclin) shown in Figure 5D. ARPE19 cells were transfected with control or Parkin siRNA and treated with Aβ in the presence or absence of HN. Phosphorylated Beclin1 levels in mitochondrial and cytosolic fractions were analyzed by immunoblotting, normalized to COX IV and GAPDH, respectively, and expressed as the mitochondria/cytosolic ratio. (B) Quantification of total Beclin1 shown in Figure 5D under the same experimental conditions and expressed as the mitochondria/cytosolic ratio. *p* values were determined using one‐way ANOVA with Tukey's multiple‐comparison test (A, B). ****p < 0.0001. Data are mean ± S.D. from biologically independent experiments (*n* = 3).
**Figure S6:** Orthogonal Z‐stack images of flat‐mounted RPE from mice subretinally injected with FITC‐labeled Aβ, with or without intravitreal HN administration. Representative confocal images correspond to the same images shown in Figure 6A, with orthogonal views (X‐Z and Y‐Z planes) reconstructed from Z‐stack images displayed to the right and bottom of the merged images, confirming intracellular localization of FITC‐labeled Aβ within the RPE. Scale bar, 10 μm.
**Figure S7:** Knockdown efficiency of siRNAs in ARPE19 cells. (A) Beclin1 siRNAs were transfected into ARPE19 cells using RNAiMAx, and Beclin1 expression level were analyzed by immunoblot analysis. (B) Parkin siRNAs were transfected into ARPE19 cells using RNAiMAx, and Parkin expression level were analyzed by immunoblot analysis. (C) PINK siRNAs were transfected into ARPE19 cells using RNAiMAx, and PINK expression level were analyzed by immunoblot analysis. *p* values were determined using unpaired two‐tailed *t*‐tests. *p < 0.05. Data are mean ± S.D. from biologically independent experiments (*n* = 3).
**Table S1:** List of pimary, conjugated, and secondary antibodies used in this study.

## Data Availability

The data that support the findings of this study are available from the corresponding author upon reasonable request.
