# Supplementary material for: Humanin Mitigates Aβ‐Induced Retinal Pigment Epithelium Injury via AMPK‐Beclin1‐Dependent Mitophagy
Source: Aging Cell. 2026 Jun 23;25(7):e70601. doi: 10.1111/acel.70601 (PMC13288060; doi:10.1111/acel.70601)
Supplement: Supplementary file 1 — Figure S1: HN enhances nuclear localization of TFEB in Aβ‐treated iPSC‐derived RPE cells. (A) Representative confocal images and quantification of iPSC‐derived RPE cells showing TFEB (green), ZO‐1 (red), and nuclei (DAPI, blue) following treatment with Aβ alone or in combination with HN. Aβ treatment resulted in reduced nuclear localization of TFEB, whereas co‐treatment with HN increased TFEB nuclear localization. (B) Quantification of nuclear TFEB signal. Scale bar, 10 μm. *p < 0.05, **p < 0.01, and ***p < 0.001. Data are mean ± S.D. from biologically independent experiments (n = 3). Figure S2: Time‐dependent changes in mitochondrial mass‐related protein expression following Aβ and HN treatment. (A) Representative immunoblot images showing the expression of mitochondrial mass‐related proteins TIM23, COX IV, and TOM20 in ARPE19 cells treated with Aβ alone or in combination with HN at 3, 6, and 24 h post‐treatment. The 6 h time point corresponds to the data presented in Figure 2B. (B) Densitometric quantification of band intensities for TIM23, COX IV, and TOM20 at each time point, normalized to GAPDH. *p < 0.05, **p < 0.01, and ***p < 0.001. Data are mean ± S.D. from biologically independent experiments (n = 3). Figure S3: Dynamics in colocalization of mitochondria with Aβ and LAMP1 by HN throughout the time course. (A) Representative confocal images and quantification of ARPE19 cells treated with FITC‐labeled Aβ alone or with HN at 3, 6, and 24 h after treatment, tracking the colocalization of FITC‐labeled Aβ and mitochondria. (B) Representative confocal images and quantification of ARPE19 cells treated with Aβ alone or with HN at 3, 6, and 24 h after treatment, tracking the colocalization of LAMP1 and mitochondria. Scale bar, 10 μm. p values were determined using one‐way ANOVA with Tukey's multiple‐comparison test (A, B). *p < 0.05 and **p < 0.01, and ***p < 0.001. Data are mean ± S.D. from biologically independent experiments (n = 5). Figure S4: HN is not involved [file ACEL-25-e70601-s001.docx]

**Supplementary Information**

**The supplementary material includes:**

**Fig. S1. HN enhances nuclear localization of TFEB in Aβ-treated iPSC-derived RPE cells.**

**Fig. S2. Time-dependent changes in mitochondrial mass-related protein expression following Aβ and HN treatment.**

**Fig. S3. Dynamics in colocalization of mitochondria with Aβ and LAMP1 by HN throughout the time course.**

**Fig. S4. HN is not involved in PINK-mediated recruitment of LAMP1 to mitochondria and clearance of Aβ-accumulated mitochondria.**

**Fig. S5. Quantification of phosphorylated Beclin1 and total Beclin1 distribution between mitochondrial and cytosolic fractions.**

**Fig. S6. Orthogonal Z-stack images of flat-mounted RPE from mice subretinally injected with FITC-labeled Aβ, with or without intravitreal HN administration.**

**Fig. S7. Knockdown efficiency of siRNAs in in ARPE19 cells.**

**Table S1. List of pimary, conjugated, and secondary antibodies used in this study**

**Figure S1**

**
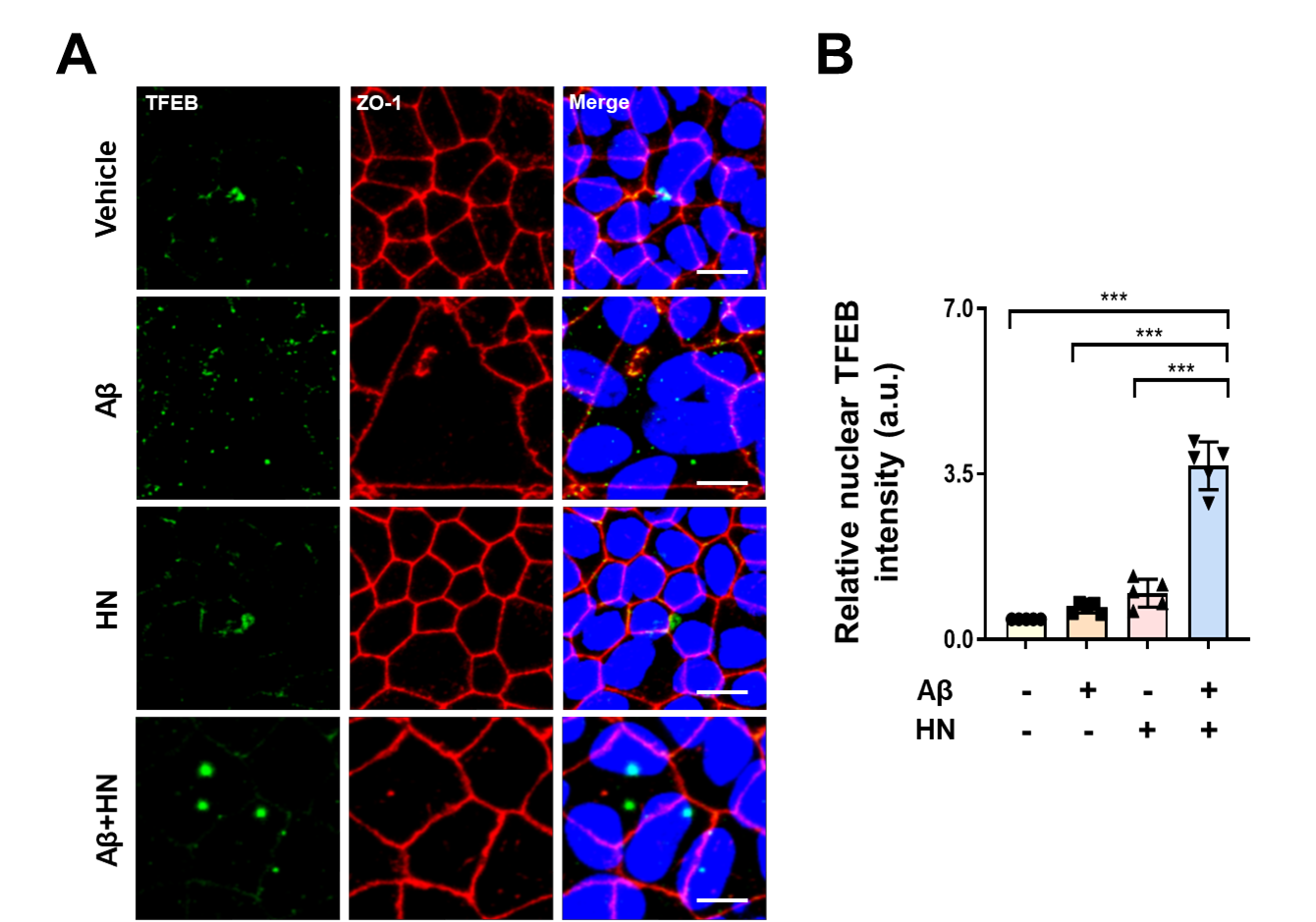
**

**Fig. S1. HN enhances nuclear localization of TFEB in Aβ-treated iPSC-derived RPE cells.** (A) Representative confocal images and quantification of iPSC-derived RPE cells showing TFEB (green), ZO-1 (red), and nuclei (DAPI, blue) following treatment with Aβ alone or in combination with HN. Aβ treatment resulted in reduced nuclear localization of TFEB, whereas co-treatment with HN increased TFEB nuclear localization. (B) Quantification of nuclear TFEB signal. Scale bar, 10 μm. **p* < 0.05, ***p* < 0.01, and ****p* < 0.001. Data are mean ± S.D. from biologically independent experiments (*n*=3).

**Figure S2**

**
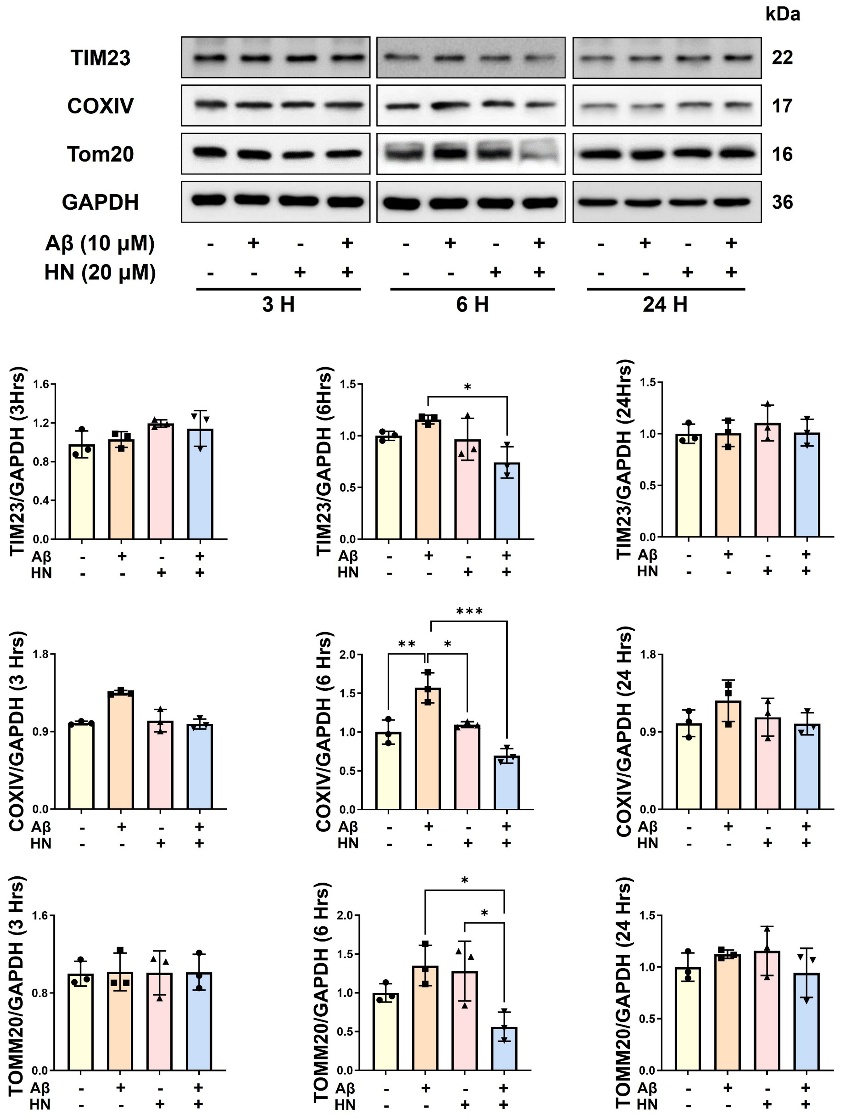
**

**Fig. S2. Time-dependent changes in mitochondrial mass-related protein expression following Aβ and HN treatment.** (A) Representative immunoblot images showing the expression of mitochondrial mass-related proteins TIM23, COX IV, and TOM20 in ARPE19 cells treated with Aβ alone or in combination with HN at 3, 6, and 24 hours post-treatment. The 6 h time point corresponds to the data presented in Fig. 2B. (B) Densitometric quantification of band intensities for TIM23, COX IV, and TOM20 at each time point, normalized to GAPDH. **p* < 0.05, ***p* < 0.01, and ****p* < 0.001. Data are mean ± S.D. from biologically independent experiments (*n*=3).

**Figure S3**

**
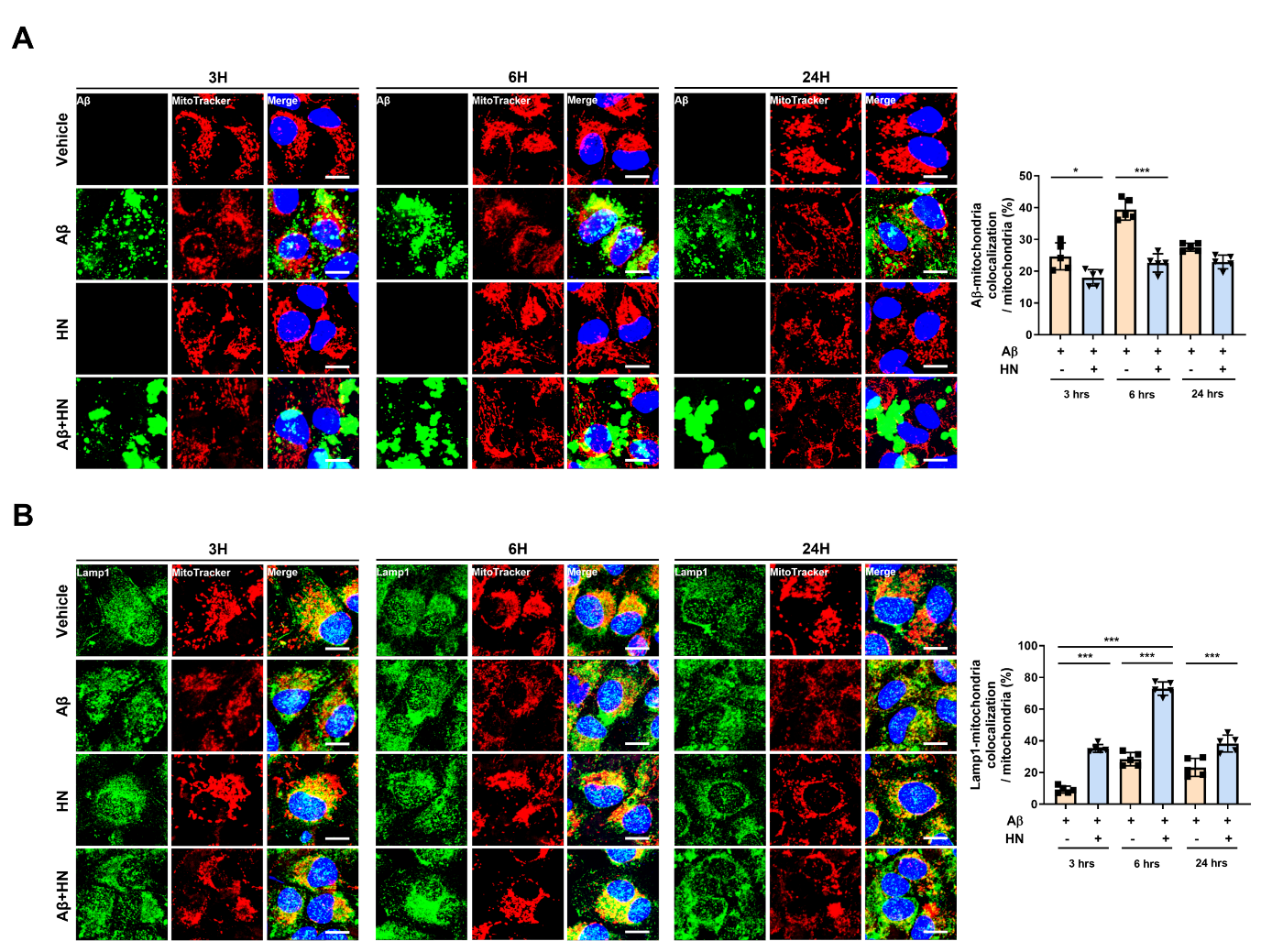
**

**Fig. S3. Dynamics in colocalization of mitochondria with Aβ and LAMP1 by HN throughout the time course. (A)** Representative confocal images and quantification of ARPE19 cells treated with FITC-labeled Aβ alone or with HN at 3, 6, and 24 hours after treatment, tracking the colocalization of FITC-labeled Aβ and mitochondria. **(B)** Representative confocal images and quantification of ARPE19 cells treated with Aβ alone or with HN at 3, 6, and 24 hours after treatment, tracking the colocalization of LAMP1 and mitochondria. Scale bar, 10 μm. *P* values were determined using one-way ANOVA with Tukey’s multiple-comparison test (A, B). **p* < 0.05 and ***p* < 0.01, and ****p* < 0.001. Data are mean ± S.D. from biologically independent experiments (*n*=5).

**Figure S4**

**
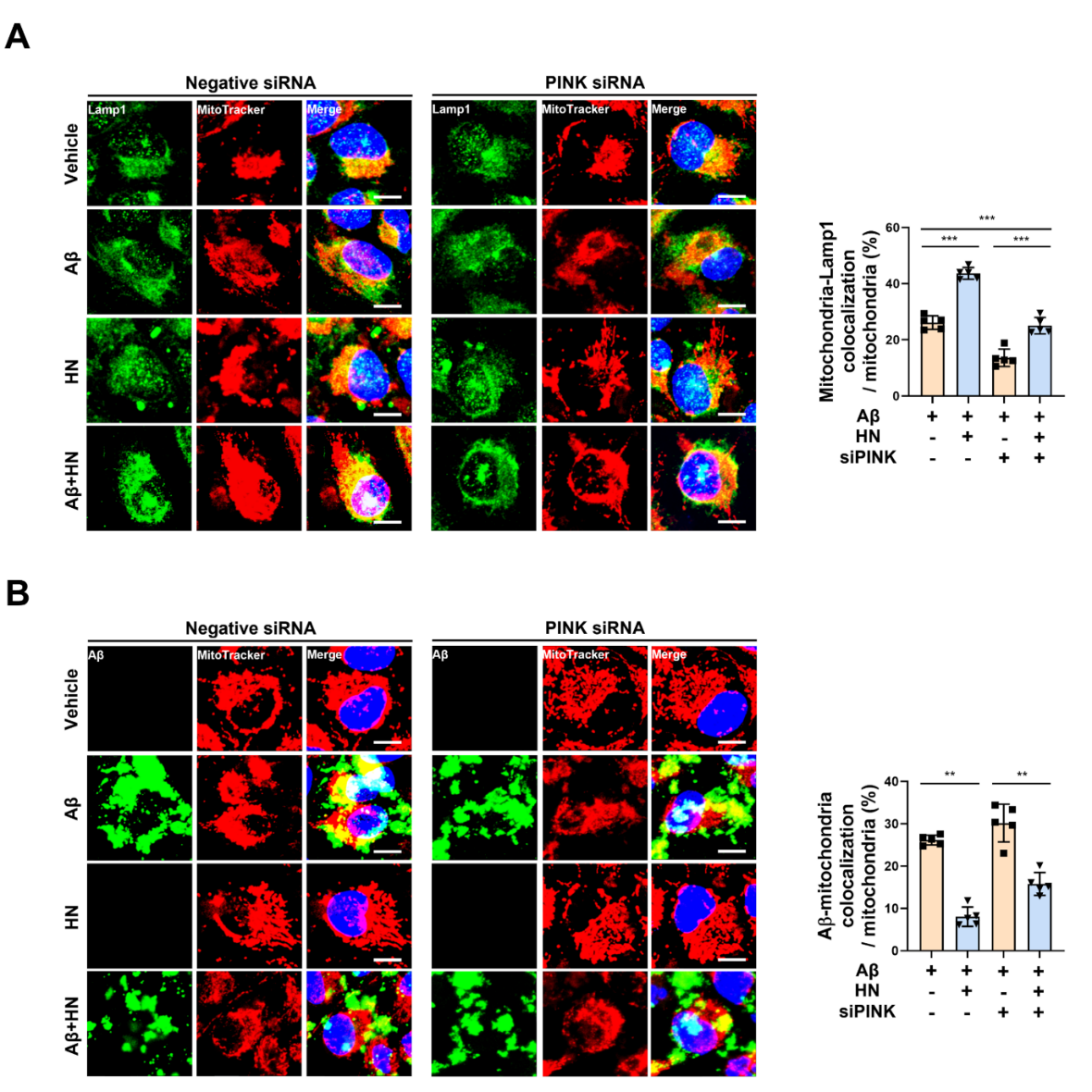
**

**Fig. S4. HN is not involved in PINK-mediated recruitment of LAMP1 to mitochondria and clearance of Aβ-accumulated mitochondria. (A)** Representative confocal images and quantification of ARPE19 cells to assess the role of PINK in the recruitment of LAMP1 to mitochondria by HN. **(B)** Representative confocal images and quantification of ARPE19 cells to evaluate the role of PINK in the clearance of Aβ-accumulated mitochondria by HN. *P* values were determined using one-way ANOVA with Tukey’s multiple-comparison test (A, B). **p* < 0.05 and ***p* < 0.01, and ****p* < 0.001. Data are mean ± S.D. from biologically independent experiments (*n*=3).

**Figure S5**

**Fig. S5. Quantification of phosphorylated Beclin1 and total Beclin1 distribution between mitochondrial and cytosolic fractions.** (A) Quantification of phosphorylated Beclin1 (p-Beclin) shown in Fig. 5D. ARPE19 cells were transfected with control or Parkin siRNA and treated with Aβ in the presence or absence of HN. Phosphorylated Beclin1 levels in mitochondrial and cytosolic fractions were analyzed by immunoblotting, normalized to COX IV and GAPDH, respectively, and expressed as the mitochondria/cytosolic ratio. (B) Quantification of total Beclin1 shown in Fig. 5D under the same experimental conditions and expressed as the mitochondria/cytosolic ratio. *P* values were determined using one-way ANOVA with Tukey’s multiple-comparison test (A, B). *****p* < 0.0001. Data are mean ± S.D. from biologically independent experiments (*n*=3).

**Figure S6**


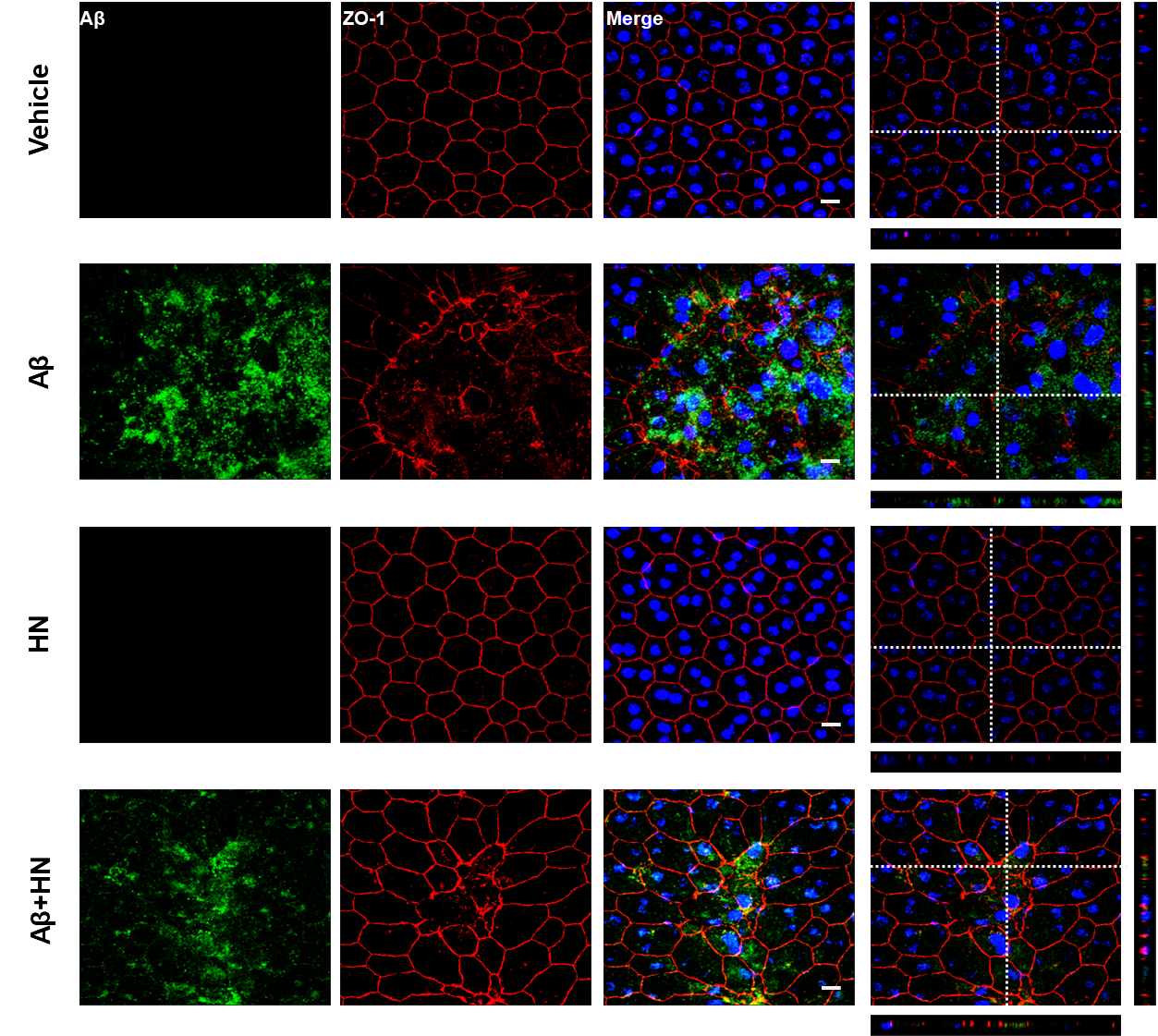


**Fig. S6. Orthogonal Z-stack images of flat-mounted RPE from mice subretinally injected with FITC-labeled Aβ, with or without intravitreal HN administration.** Representative confocal images correspond to the same images shown in Figure 6A, with orthogonal views (X-Z and Y-Z planes) reconstructed from Z-stack images displayed to the right and bottom of the merged images, confirming intracellular localization of FITC-labeled Aβ within the RPE. Scale bar, 10 μm.

**Figure S7**

**
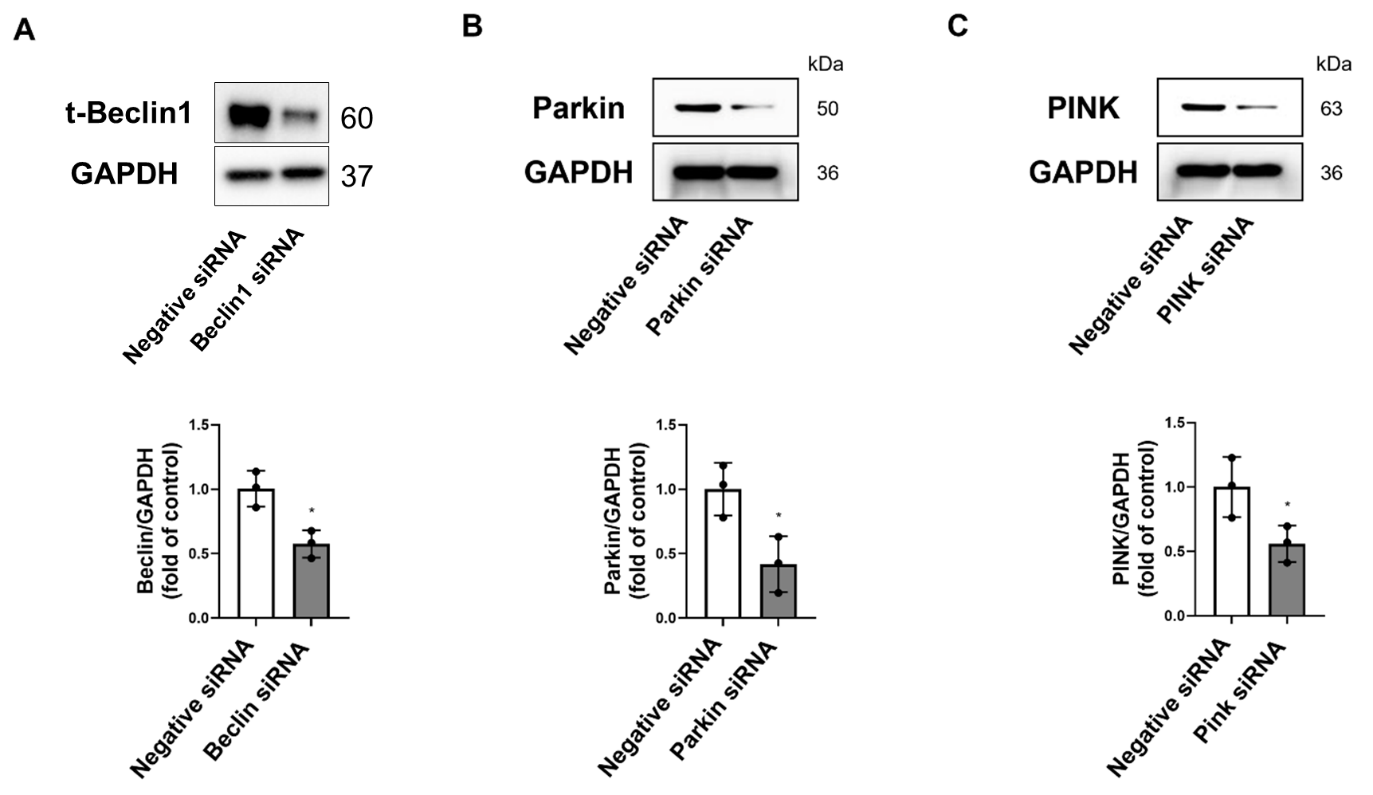
**

**Fig. S7. Knockdown efficiency of siRNAs in ARPE19 cells. (A)** Beclin1 siRNAs were transfected into ARPE19 cells using RNAiMAx, and Beclin1 expression level were analyzed by immunoblot analysis. **(B)** Parkin siRNAs were transfected into ARPE19 cells using RNAiMAx, and Parkin expression level were analyzed by immunoblot analysis. **(C)** PINK siRNAs were transfected into ARPE19 cells using RNAiMAx, and PINK expression level were analyzed by immunoblot analysis. *P* values were determined using unpaired two-tailed *t*-tests. **p* < 0.05. Data are mean ± S.D. from biologically independent experiments (*n*=3).

**Table S1. List of pimary, conjugated, and secondary antibodies used in this study.**

| **Antibody** | **Company** | **Catalog No.** | **RRID** | **Dilution** |
| --- | --- | --- | --- | --- |
| ZO-1 | Cell Signaling Technology | 13663 | AB_2798287 | 1:100 (IF) |
| LAMP1 | Invitrogen | PA1-654A | AB_2134611 | 1:100 (IF)  1:1000 (WB) |
| Parkin | Invitrogen | 39-0900 | AB_2533396 | 1:100 (IF)  1:1000 (IP) |
| Parkin | Cell Signaling Technology | 2143 | AB_823625 | 1:1000 (IP) |
| Beclin1 | Invitrogen | PA5-96649 | AB_2808451 | 1:100 (IF)  1:1000 (IP) |
| Beclin1 | Cell Signaling Technology | 4122 | AB_11178656 | 1:1000 (IP) |
| TOM20 | Cell Signaling Technology | 42406 | AB_2687663 | 1:100 (IF) |
| Alexa Fluor™ 594 ZO-1 | Invitrogen | 339194 | AB_2532188 | 1:200 (IF) |
| Goat anti-Rabbit IgG(H+L), Alexa Fluor 647 | Invitrogen | A48265 | AB_2895299 | 1:200 (IF) |
| Goat anti-Mouse IgG(H+L), Alexa Fluor 488 | Invitrogen | A32723 | AB_2633275 | 1:200 (IF) |
| Goat anti-Rabbit IgG(H+L), Alexa Fluor 488 | Invitrogen | A32731 | AB_2633280 | 1:200 (IF) |
| Goat anti-Rabbit IgG(H+L), Alexa Fluor 594 | Invitrogen | A32740 | AB_2762824 | 1:200 (IF) |
| PINK1 | Cell Signaling Technology | 6946 | AB_11179069 | 1:1000 (IP) |
| PINK1 | Santa Cruz Biotechnology | sc-518052 | AB_2861352 | 1:500 (IP) |
| Anti-rabbit 2° Ab, HRP conjugate | Cell Signaling Technology | 7074 | AB_2099233 | 1:2000 (WB) |
| Anti-mouse 2° Ab, HRP conjugate | Cell Signaling Technology | 7076 | AB_330924 | 1:2000 (WB) |
| Amyloid β | Cell Signaling Technology | 8243 | AB_2797642 | 1:1000 (WB) |
| TFEB | Cell Signaling Technology | 4240 | AB_11220225 | 1:1000 (WB) |
| phospho TFEB (ser142) | Sigma-Aldrich | ABE1971 | AB_2928101 | 1:1000 (WB) |
| SQSTM1/p62 | Cell Signaling Technology | 8025 | AB_10859911 | 1:1000 (WB) |
| LC3B | Cell Signaling Technology | 3868 | AB_2137707 | 1:1000 (WB) |
| GAPDH | Cell Signaling Technology | 2118 | AB_561053 | 1:1000 (WB) |
| TIM23 | Santa Cruz Biotechnology | sc-514463 | AB_2923126 | 1:500 (WB) |
| COX IV | Abcam | ab33985 | AB_879754 | 1:500 (WB) |
| TOM20 | Cell Signaling Technology | 42406 | AB_2687663 | 1:1000 (WB) |
| AMPKα | Cell Signaling Technology | 2532 | AB_330331 | 1:1000 (WB) |
| Phospho-AMPKα | Cell Signaling Technology | 2535 | AB_331250 | 1:1000 (WB) |
| ULK1 | Cell Signaling Technology | 8054 | AB_11178668 | 1:1000 (WB) |
| Phospho-ULK1 | Cell Signaling Technology | 14202 | AB_2665508 | 1:1000 (WB) |
| Beclin1 | Cell Signaling Technology | 3738 | AB_490837 | 1:1000 (WB) |
| Phospho-Beclin1 | Abbiotec | 254515 | AB_3068331 | 1:1000 (WB) |
| Acetyl-CoA Carboxylase | Cell Signaling Technology | 3676 | AB_2219397 | 1:1000 (WB) |
| Phospho-Acetyl-CoA Carboxylase | Cell Signaling Technology | 3661 | AB_330337 | 1:1000 (WB) |
| PINK1 | Cell Signaling Technology | 6946 | AB_11179069 | 1:1000 (WB) |
| Beclin1 | Cell Signaling Technology | 3495 | AB_1903911 | 1:1000 (WB) |
| Parkin | Cell Signaling Technology | 4211 | AB_2159920 | 1:1000 (WB) |
| ZO-1 | Cell Signaling Technology | 13663 | AB_2798287 | 1:1000 (WB) |
| F-actin | Abcam | ab205 | AB_302794 | 1:500 (WB) |
